# NIMA-related kinases redundantly regulate vegetative and reproductive development in *Arabidopsis thaliana*

**DOI:** 10.1093/pcp/pcag056

**Published:** 2026-04-28

**Authors:** Shogo Takatani, Mai Kanazawa, Kotaro Sakamoto, Yuki Tomita, Nozomi Kawamoto, Tatsuya Sakai, Takashi Araki, Hiroyasu Motose

**Affiliations:** Graduate School of Environmental, Life, Natural Science and Technology, Okayama University, Okayama 700-8530, Japan; Department of Biology, Faculty of Science, Okayama University, Tsushimanaka 3-1-1, Okayama 700-8530, Japan; Department of Biology, Faculty of Science, Okayama University, Tsushimanaka 3-1-1, Okayama 700-8530, Japan; Graduate School of Biostudies, Division of Integrated Life Science, Kyoto University, Sakyo-ku, Kyoto 606-8501, Japan; Graduate School of Biostudies, Division of Integrated Life Science, Kyoto University, Sakyo-ku, Kyoto 606-8501, Japan; Graduate School of Science and Technology, Niigata University, Nishi-ku, Niigata 950-2181, Japan; Graduate School of Biostudies, Division of Integrated Life Science, Kyoto University, Sakyo-ku, Kyoto 606-8501, Japan; Graduate School of Environmental, Life, Natural Science and Technology, Okayama University, Okayama 700-8530, Japan; Department of Biology, Faculty of Science, Okayama University, Tsushimanaka 3-1-1, Okayama 700-8530, Japan

**Keywords:** *Arabidopsis thaliana*, flowering, FLOWERING LOCUS T, NIMA-related kinase, organ development, tubulin

## Abstract

Never-in-mitosis A-related kinases (NEKs) are conserved protein kinases in eukaryotes that regulate cell division and elongation. In *Arabidopsis thaliana*, NEK6 has been shown to control anisotropic growth through cortical microtubule depolymerization, but the functions of other NEK family members remain largely unknown. Here, we show that *Arabidopsis* NEKs redundantly regulate organ growth and flowering through phosphorylation-dependent mechanisms. Expression analyses revealed overlapping promoter activities of *NEK* genes in meristems, vascular tissues, and floral organs. While most single mutants showed no obvious phenotype, multiple mutants exhibited defects in root growth direction, leaf elongation, vascular formation, and floral transition. Biochemical analysis showed that NEK3 and NEK6 phosphorylate both β-tubulin and γ-tubulin, indicating a conserved role in microtubule regulation. In addition, several NEKs interacted with the florigen FLOWERING LOCUS T (FT), and NEK3 phosphorylated FT *in vitro* and inhibited the FT–FD interaction in transient assays, suggesting a role in flowering regulation. These findings show that *Arabidopsis* NEKs redundantly coordinate vegetative and reproductive development through phosphorylation-dependent regulation of microtubules and the flowering pathway.

## Introduction

Never-in-mitosis A-related kinases (NEKs) are conserved serine/threonine protein kinases found in most eukaryotes. The founding member, Never in mitosis A, was identified in the filamentous fungus *Aspergillus nidulans* as an essential regulator of the G2/M transition (Oakley and Morris 1983, Osmani et al. 1987, 1988). In fungi and animals, NEKs control multiple aspects of cell division, including centrosome separation, spindle formation, and cytokinesis, and many NEKs localize to microtubule-organizing centers or mitotic structures (O'Connell et al. 2003, Fry et al. 2012). In addition to their roles in mitosis, several NEKs regulate microtubule-based structures such as cilia and flagella (Quarmby and Mahjoub 2005, Parker et al. 2007), indicating that this kinase family mainly controls microtubule-dependent processes across eukaryotes.

Compared with fungi and animals, the functions of plant NEKs remain poorly understood. In the unicellular green alga *Chlamydomonas reinhardtii*, NEK proteins regulate flagellar disassembly and cell cycle progression (Mahjoub et al. 2002, Bradley and Quarmby 2005, Meng and Pan 2016), suggesting conserved roles in the regulation of microtubules and cell division. In flowering plants, several NEKs have been implicated in reproductive development (Zhang et al. 1996, Pnueli et al. 2001, Cloutier et al. 2005, Fujii et al. 2009). For example, a tomato NEK named “SPAK” (SP-associated kinase) interacts with the TERMINAL FLOWER1 (TFL1)/CENTRORADIALIS (CEN)/SELF-PRUNING (SP) family and 14-3-3 proteins, suggesting a possible link between NEKs and inflorescence development (Pnueli et al. 2001). However, most of these studies relied on overexpression or protein interaction assays, and the physiological functions of plant NEKs remain largely unclear.

The genome of *Arabidopsis thaliana* encodes seven NEK members (NEK1–NEK7), all of which contain a conserved N-terminal kinase domain but differ in their C-terminal regulatory regions (Cloutier et al. 2005, Vigneault et al. 2007, Fig. 1). Previous studies suggested that *Arabidopsis* NEKs participate in plant morphogenesis (Motose et al. 2008, Sakai et al. 2008, Agueci et al. 2012, Takatani et al. 2017, 2020, Yang et al. 2024) and stress responses (Lee et al. 2010, Ning et al. 2011, Zhang et al. 2011, Pan et al. 2017, reviewed in Takatani et al. 2015). However, their individual roles and possible redundancy have not been systematically examined. Among these members, NEK6 has been studied most extensively and functions as a regulator of anisotropic cell growth. Its loss of function causes abnormal growth direction of epidermal cells (Motose et al. 2008, Sakai et al. 2008) and wavy growth of hypocotyls and roots (Motose et al. 2012, Takatani et al. 2020). NEK6 localizes to cortical microtubules and phosphorylates β-tubulin to promote the removal of aberrant microtubules, thereby contributing to the control of directional growth (Motose et al. 2011, Takatani et al. 2017, 2020). Consistent with this role, NEK-dependent regulation of microtubules is conserved in early-diverging land plants such as *Marchantia polymorpha*, in which NEK controls the tip growth of rhizoids (Otani et al. 2018, Mase et al. 2022, 2025).

**Figure 1:**
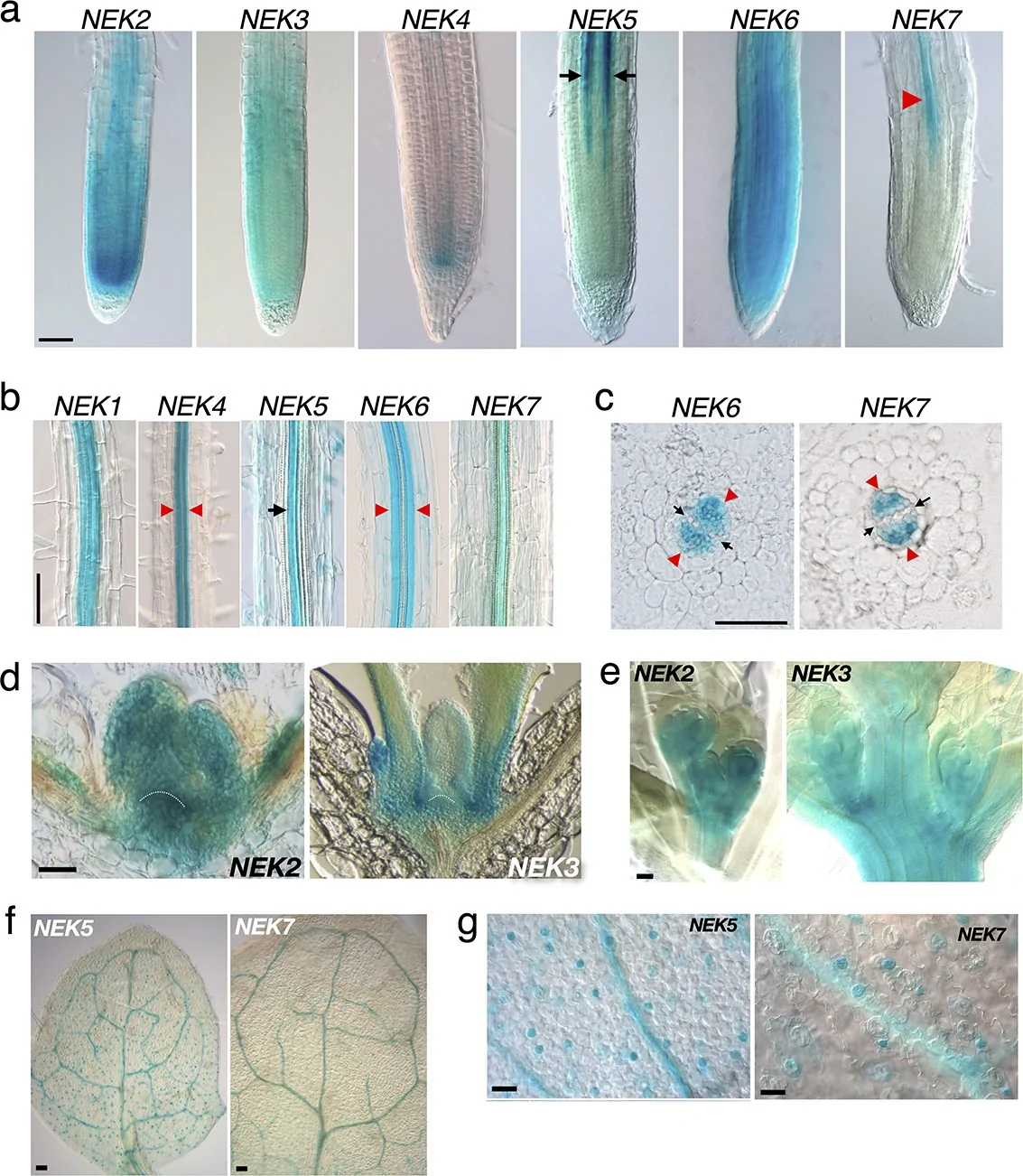
The promoter activity of *NEK* genes. Histochemical GUS staining of *NEK* promoter–GUS fusions in root tips (a), root vascular tissues (b), root cross-sections (c), cross-sections of the shoot apex (d), inflorescences (e), leaves (f), and guard cells (g). Black arrow: xylem; red arrowhead: phloem. Dotted lines in (d) show the shoot apical meristem. Bars = 50 *μ*m (a, c, d, f, and g) and 100 *μ*m (b, e).

Although NEK6 has been characterized as a key regulator of microtubule-dependent directional growth, the functions of other NEK family members remain largely unknown. Several NEKs show overlapping expression patterns, and single mutants often display weak or no visible phenotypes, suggesting that functional redundancy may obscure their roles. In addition, it is unclear whether plant NEKs share common molecular targets, such as tubulin, or whether they regulate plant development through distinct pathways. Furthermore, previous reports suggesting links between NEKs and flowering regulators raise the possibility that NEKs may control floral transition, but this hypothesis has not been tested genetically.

In this study, we performed a comprehensive analysis of the *Arabidopsis* NEK family to clarify the redundant and distinct functions of NEKs in plant development. We examined phylogenetic relationships, expression patterns, and mutant phenotypes of all seven NEK genes, and analyzed multiple mutants to uncover their hidden functions. Our results demonstrate that *Arabidopsis* NEKs redundantly regulate root growth direction, leaf elongation, and flowering through a phosphorylation-dependent mechanism.

## Results

### Characterization of the plant NEK family

To understand the evolutionary relationships of plant NEKs, We first constructed a phylogenetic tree using the conserved kinase domains of NEK proteins from representative plant species (Supplementary Fig. S1). Algal NEKs were relatively divergent in both sequence and domain structure, whereas land plant NEKs formed a clear monophyletic clade composed of closely related subgroups (Supplementary Fig. S1a). Among the five NEKs of *Klebsormidium nitens*, KnNEK5 was most closely related to land plant NEKs (Supplementary Fig. S1c). Notably, charophycean algae contained NEKs with structural features similar to those of land plant NEKs, including a conserved N-terminal kinase domain and a long C-terminal regulatory tail. These charophycean NEKs have a CAP-Gly (cytoskeleton-associated protein-glycine-rich) microtubule-binding domain in the C-terminal tail, suggesting that the ancestral form of land plant NEKs already possessed microtubule-related functions.

Land plant NEKs were divided into two major groups, AtNEK1234-type and AtNEK567-type, according to the *Arabidopsis* members. The AtNEK1234 group consisted of angiosperm NEKs, whereas the AtNEK567 group included NEKs of bryophytes, lycophytes, monilophytes, gymnosperms, and angiosperms. The AtNEK567 group could be further subdivided into three subgroups corresponding to AtNEK6-like, AtNEK5-like, and AtNEK7-like genes. The AtNEK6 subgroup was widely conserved in land plants, whereas the AtNEK5 subgroup was restricted to seed plants. Interestingly, the AtNEK7 subgroup was found only in Brassicales, suggesting lineage-specific diversification and Brassicales-related function.

These phylogenetic relationships indicate that land plant NEKs likely originated from a single ancestral kinase related to the AtNEK6 subgroup and subsequently diversified into several lineage-specific subgroups (i.e. angiosperm AtNEK1234-type, seed plant AtNEK5-like genes, and the Brassicales-specific AtNEK7 subgroup). To obtain clues to the functional diversification of these genes, we next examined the expression patterns of *Arabidopsis* NEK family members.

### Expression patterns of *Arabidopsis NEK* genes

To investigate possible functional differences among *NEK* genes, we examined their expression patterns by RT-PCR (Supplementary Fig. S1d) and promoter–reporter lines (Figs. 1 and 2 and Supplementary Fig. S2). Reverse Transcription-PCR (RT-PCR) analysis showed that all seven *NEK* genes were expressed in multiple organs, although each gene exhibited a characteristic pattern of transcript accumulation (Supplementary Fig. S1d). *NEK1* and *NEK2* exhibited similar expression profiles, with relatively high levels in hypocotyls and flowers, whereas *NEK3* was enriched in flowers, *NEK4* in hypocotyls, *NEK5* in hypocotyls and stems, *NEK6* in stems and flowers, and *NEK7* in roots.

**Figure 2:**
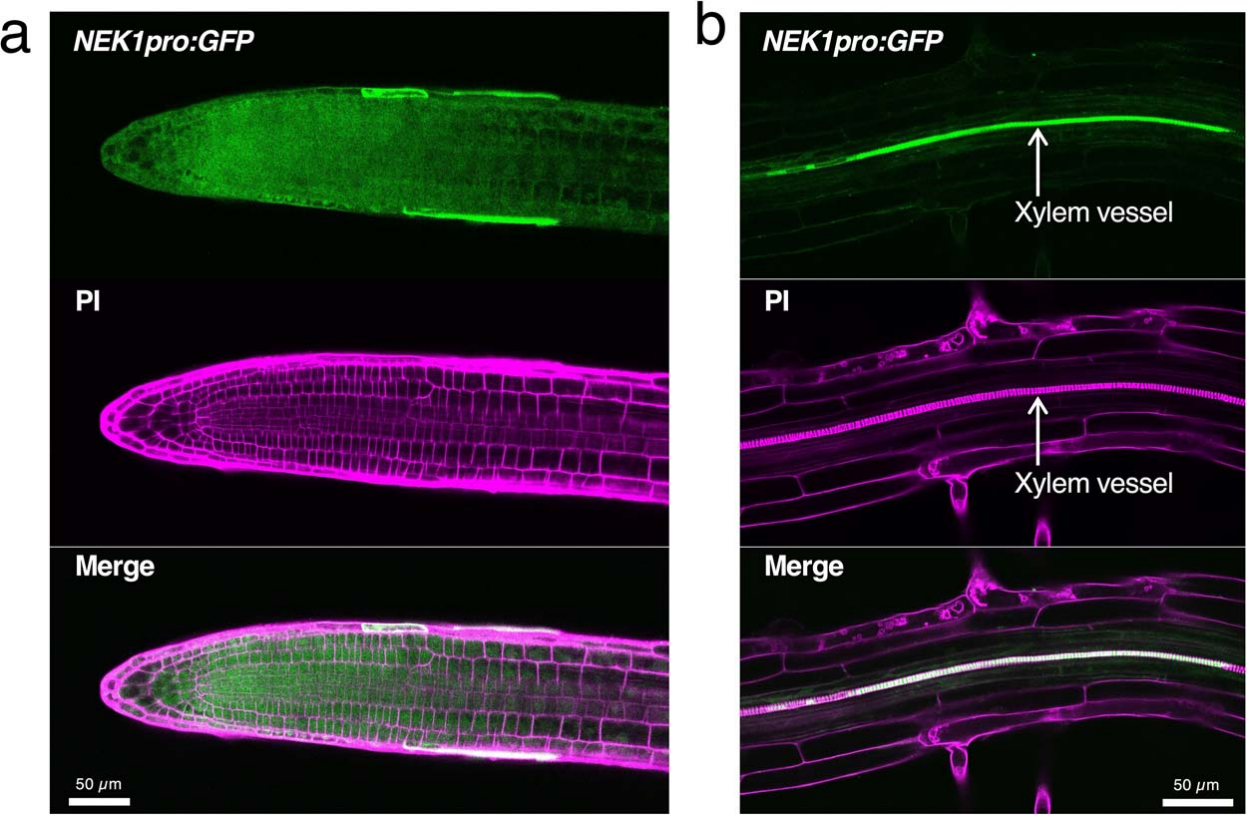
The promoter activity in roots of *NEK1* promoter–GFP fusion. The roots of transgenic plants harboring *NEK1* promoter–GFP fusion were observed under a confocal laser-scanning microscope. The GFP signal is shown in green; cell walls were stained by PI (magenta). Root tip (a) and xylem vessel (b). GFP signal is observed in root meristem (a), lateral root cap (a), and xylem vessel (b). Bars = 50 *μ*m.

To determine spatial expression patterns, we generated transgenic plants expressing β-glucuronidase (GUS) or green fluorescent protein (GFP) under the control of each *NEK* promoter (Figs. 1 and 2 and Supplementary Fig. S2). We analyzed at least three independent transgenic lines for each construct by histochemical GUS staining or confocal microscopy. In roots, promoter activity of *NEK2, 3, 4*, and *6* was detected in the root apical meristem (Fig. 1a). NEK4 promoter activity was restricted in the area around the quiescent center and in the procambium, whereas *NEK1, 4, 5, 6*, and *7* promoters drove expression in vascular tissues (Fig. 1b). Transverse sections revealed strong activity of *NEK6* and *NEK7* in the cambium and phloem (Fig. 1c). Although we could not obtain clear sectional images of *NEK1, 4*, and *5, NEK5-GUS* showed the typical staining in two-protoxylem strands (Fig. 1a) and the metaxylem between them (Fig. 1b). *NEK4-GUS* showed staining in phloem strands similar to those in *NEK6-GUS* (Fig. 1b). *NEK1* promoter activity was also observed in the root meristem, xylem vessels, and mature cells of the lateral root cap in NEK1–GFP lines (Fig. 2). During lateral root formation, the promoters of *NEK1, 2, 3*, and *6* were active in lateral root primordia, whereas those of *NEK5* and *7* were activated after the emergence of lateral roots (Supplementary Fig. S2a). In summary, our results show the preferential promoter activities of *NEK1, 2, 3, 4*, and *6* in root meristem; *NEK4, 6*, and *7* in the phloem; and *NEK1* and *5* in xylem.

In the shoot apex, *NEK2, 3*, and *6* promoters were active in the shoot apical meristem and leaf primordia (Fig. 1d and Supplementary Fig. S2b). *NEK2* expression was particularly strong in the meristem and leaf primordia, whereas NEK3 was enriched in the outermost cell layer of leaf primordia (Fig. 1d). *NEK2, 3*, and *6* promoters were also active in inflorescences and floral primordia (Fig. 1e and Supplementary Fig. S2c), suggesting possible roles in reproductive development.

In leaves, *NEK1, 5*, and *7* promoters were active in vascular tissues, whereas *NEK2-GUS* expression was observed in the whole of developing leaves (Fig. 1f and Supplementary Fig. S2d). The promoters of *NEK5, 6*, and *7* were active in guard cells and their precursors (Fig. 1g and Supplementary Fig. S2e).

To examine subcellular localization, we generated transgenic lines expressing NEK-GFP fusions driven by a native promoter. We previously showed that NEK6-GFP was localized in cortical microtubules and mitotic apparatus such as the preprophase band, spindle, and phragmoplast (Motose et al. 2011, Takatani et al. 2017). Furthermore, transient expression of NEKs in tobacco leaves showed that all the NEKs were colocalized with NEK6 (Motose et al. 2011). In this study, we obtained NEK5-GFP transgenic lines with obvious fluorescent signals (Fig. 3). NEK5-GFP was expressed in xylem, columella, root hair, leaf vasculature, and guard cell lineage. Close-up observation showed filamentous and punctate localization of NEK5-GFP in xylem vessels, root hairs, and guard cells, suggesting microtubule localization of NEK5 in these specialized cells.

**Figure 3:**
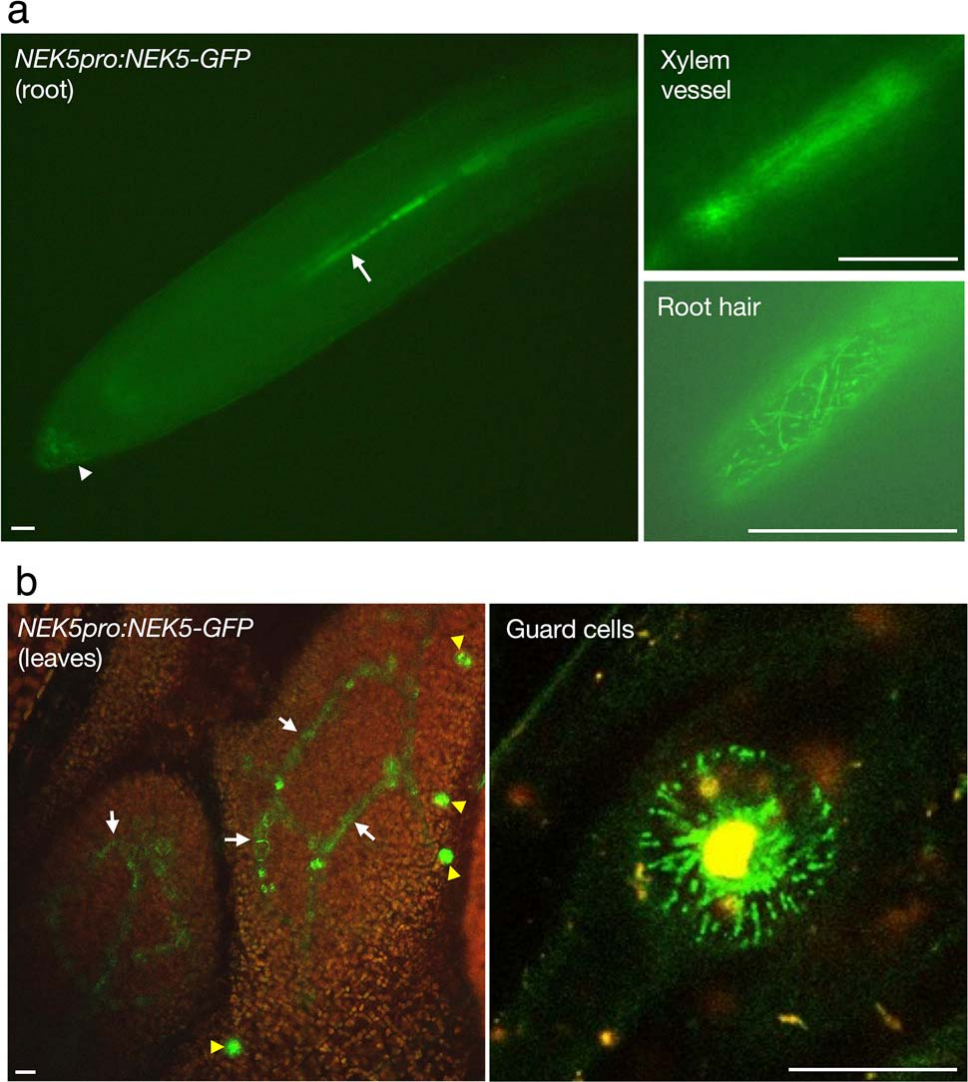
The expression and subcellular localization of NEK5-GFP fusion plants. The transgenic plants expressing NEK5-GFP fusion protein under the control of the *NEK5* promoter were observed under a confocal laser-scanning microscope. The GFP signal is shown in green and chlorophyll autofluorescence is shown in red. (a) Root tip, arrow: xylem, arrowhead: columella. (b) Leaf vasculature (white arrow) and guard cell lineage (yellow arrowhead). Bars = 20 *μ*m.

The partially overlapping but distinct expression patterns suggested that NEK genes may have both specialized and redundant functions in plant development. Because several NEK genes were coexpressed in meristems, vascular tissues, and reproductive organs, we expected functional redundancy among family members. To test this possibility, we analyzed loss-of-function mutants of NEK genes.

### Defects in root growth of *nek* mutants

To investigate the functions of NEK genes, we obtained T-DNA insertion mutants for each gene and characterized their phenotypes (Figs. 4–7 and Supplementary Fig. S3). We identified homozygous single mutants by PCR using gene-specific primers. None of them showed obvious defects in overall growth and morphogenesis, except for *nek6* mutants. Because overlapping expression suggested redundancy, we generated multiple mutant combinations and analyzed their phenotypes.

**Figure 4:**
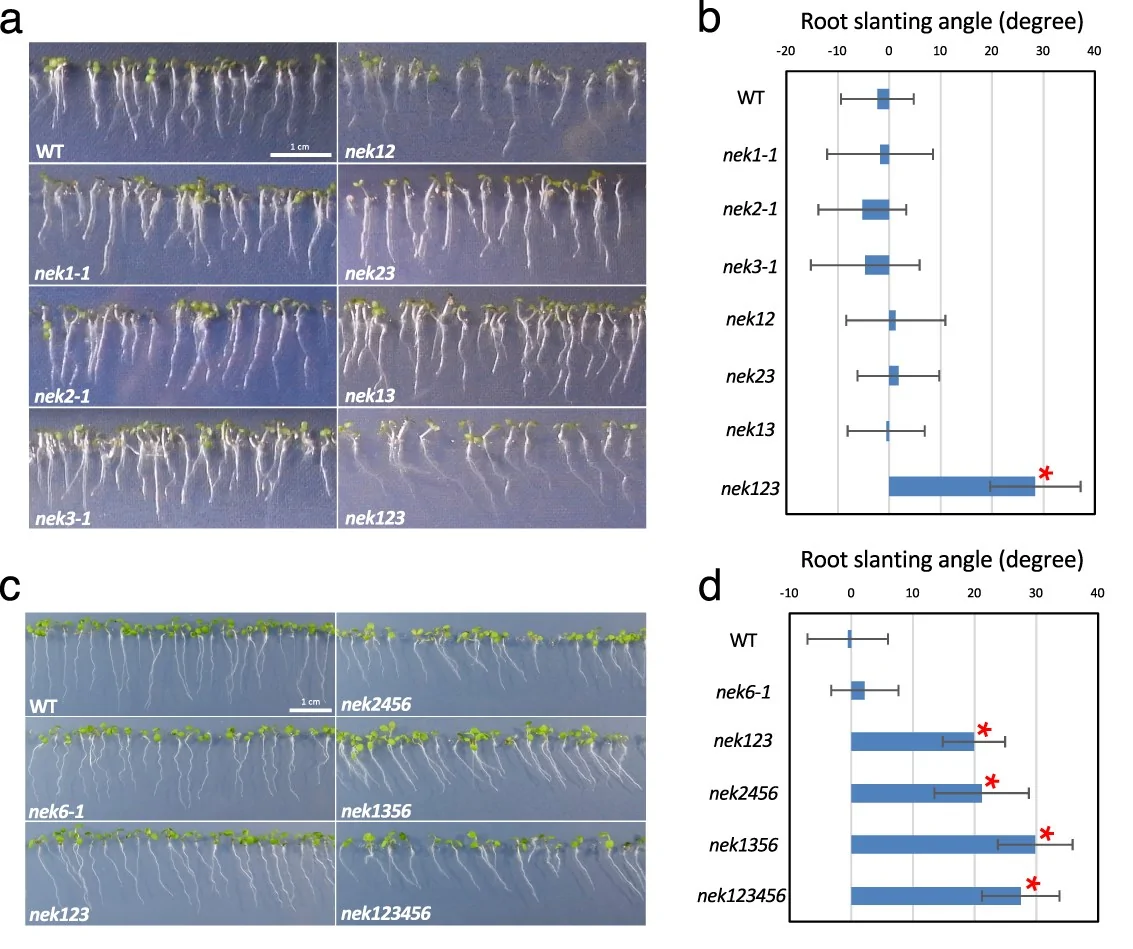
The *nek* multiple mutants show aberrant growth direction of roots. The WT and the *nek* single and multiple mutants were grown vertically on the agar medium. (a and c) The WT and the single and multiple mutants of *NEKs* were grown vertically on the agar medium for 5 days. Bars = 1 cm. (b and d) Quantification of root slanting angle of 5-day-old seedlings as grown in (a and c). Values are mean ± SD. Asterisks show significant difference from the values in the WT [Dunnett’s test, *P* < 0.0001, *n* = 14–20 plants in (b) and 20–21 plants in (d)].

**Figure 5:**
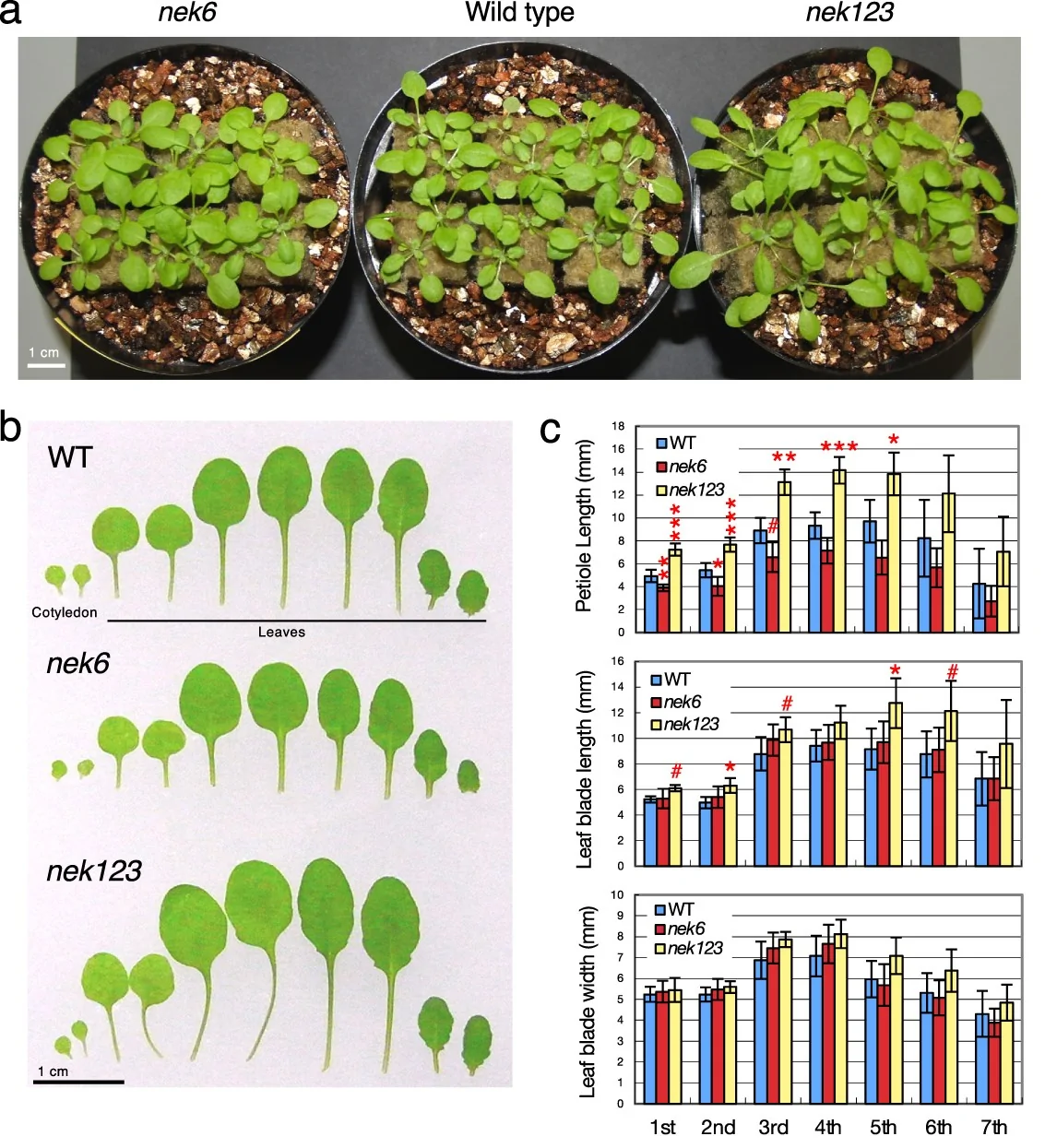
Phenotypes of the *nek6* and *nek1 nek2 nek3* triple mutants in leaf growth. (a) The morphology of the WT, *nek6* mutant, and *nek1 nek2 nek3* triple mutants grown on the soil for two weeks. (b) The growth and morphology of cotyledons and leaves of the WT, *nek6* mutant, and *nek1 nek2 nek3* triple mutants. (c) Quantification of the length of leaf petioles and blades and the width of leaf blades. The marked values show significant difference from the WT values (Dunnett’s test, ^***^P < 0.001, ^**^P < 0.01, ^*^P < 0.02, ^#^*P* < 0.05, *n* = 5 plants). Bars = 1 cm.

**Figure 6:**
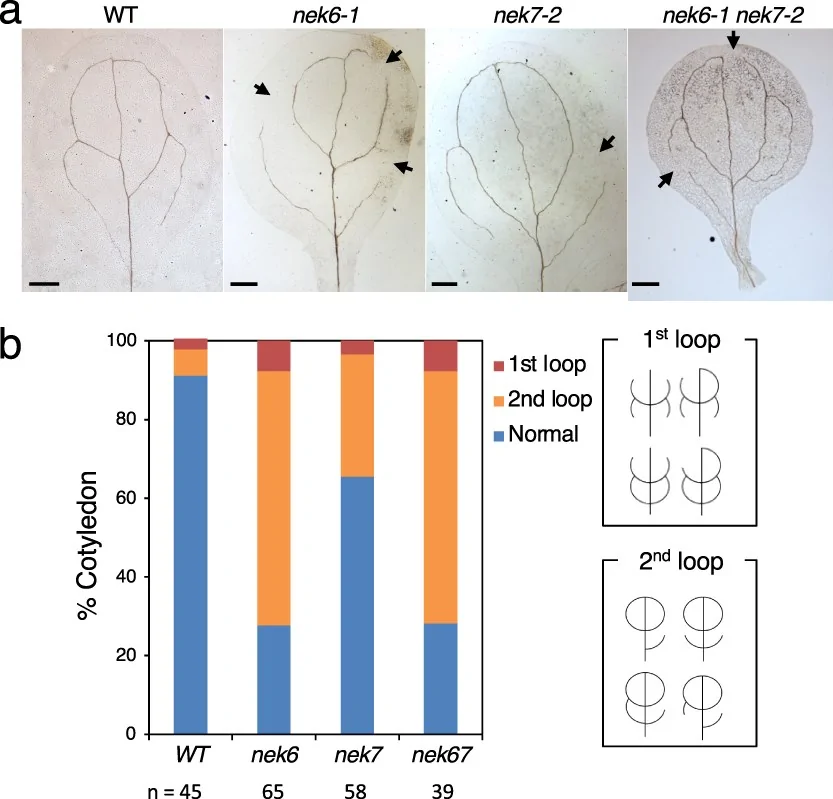
The *nek6* and *nek7* mutants show aberrant vasculature. (a) The vasculature of cotyledons of the WT and *nek6-1* and *nek7-2* mutants grown on the agar medium for 7 days. Bars = 400 *μ*m. (b) Quantification of vascular patterns of cotyledons of the WT and *nek6* and *nek7* mutants. The right-hand panels show representative discontinuous vasculatures in the first and second loop.

**Figure 7:**
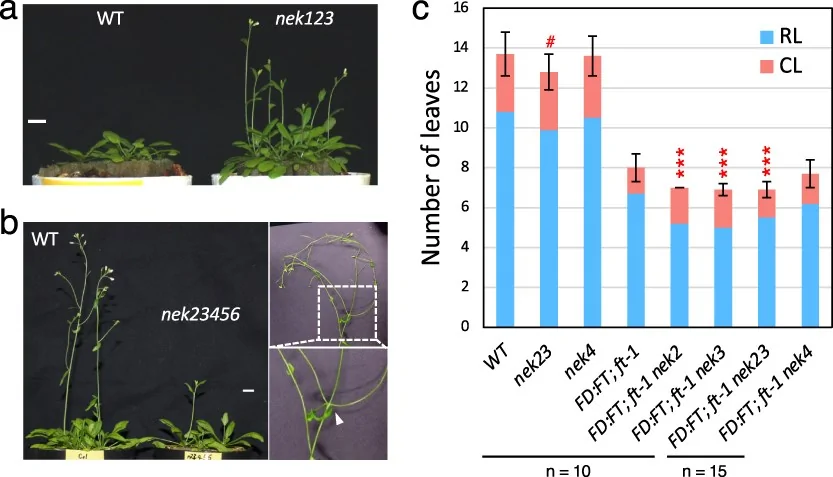
Phenotypes of *nek* multiple mutants in reproductive development. (a) The morphology of the WT and the *nek1 nek2 nek3* triple mutant grown on soil for 3 weeks. Bar = 10 mm. (b) The morphology of the WT and the *nek23456* quintuple mutant were grown on soil for 3 weeks. The arrowhead indicates two lateral inflorescent stems branched at the same position. Bar = 10 mm. (c) Quantification of the number of leaves of the WT and the *nek* mutant with or without *FD:FT ft-1* background grown on the soil for 3 weeks. Values are mean ± SD. The marked values show significant difference from the number of rosette leaves of the WT or *FD:FT ft-1* (Dunnett's test, ^***^P < 0.001, ^#^*P* < 0.05, *n* = 10, or 15 plants). CL: cauline leaf.

Although double mutants of *NEK1, NEK2*, and *NEK3* showed no obvious phenotype, the *nek1-1 nek2-1 nek3-1* triple mutant (*nek123*) exhibited abnormal root growth direction when grown on vertical agar plates (Fig. 4a and b). Roots of *nek123* consistently slanted toward the right side as observed from above, a phenotype reminiscent of mutants defective in microtubule organization (Furutani et al. 2000, Buschmann et al. 2004, Sedbrook et al. 2004, Ishida et al. 2007, Nakamura and Hashimoto 2009). The phenotype became more pronounced in quadruple and sextuple mutants [*nek2-1 nek4-1 nek5-2 nek6-4* (*nek2456*), *nek1-1 nek3-1 nek5-1 nek6-4* (*nek1356*), and *nek1-1 nek2-1 nek3-1 nek4-1 nek5-2 nek6-4* (*nek123456*)] (Fig. 4c and d).

Previous studies have shown that the root-slanting phenotype is attributed to the helical cell growth and skewing of cell files (e.g. Furutani et al. 2000, Thitamadee et al. 2002). To determine whether *nek* multiple mutants have the same defect, we examined root cell morphology by confocal microscopy after propidium iodide staining (Supplementary Fig. S4). In contrast to typical microtubule mutants showing helical cell files, *nek* multiple mutants exhibited disorganized alignment of epidermal cell files and reduced anisotropic elongation of root cells. These observations suggest that *NEK* genes redundantly regulate directional cell growth through a mechanism distinct from previously characterized microtubule regulators.

### Growth defects of leaves of *nek* mutants

Because several *NEK* genes were expressed in developing leaves, we next examined leaf growth in *nek* mutants (Fig. 5). The *nek6-1* single mutant showed reduced leaf growth as previously reported (Zhang et al. 2011, Motose et al. 2012), whereas the *nek123* triple mutant exhibited elongated leaves compared with the wild-type (WT) (Fig. 5a and b). Quantitative analysis indicated that both petiole length and leaf blade length were significantly increased in the *nek123* triple mutant, while leaf blade width was not markedly affected (Fig. 5c). In contrast, the *nek6* mutant showed shorter petioles, particularly in the lower mature leaves (first to third leaves). This short petiole phenotype was partially recovered by the expression of NEK6-GUS or NEK6-GFP fusion protein under its own promoter (Supplementary Fig. S5). Furthermore, both the number and length of epidermal cells were reduced in the petioles of *nek6*, whereas the cell number was significantly increased in those of *nek123* (Supplementary Fig. S6). These results suggest that NEK6 promotes petiole growth via cell division and elongation, whereas NEK1, 2, and 3 act antagonistically to suppress leaf elongation probably through cell division. Because microtubules regulate cell division and anisotropic growth, these phenotypes are consistent with the defects in directional growth observed in the roots of *nek* mutants.

### Aberrant vasculature of *nek* mutants

The strong promoter activities of several *NEK* genes in vascular tissues prompted us to examine whether NEK mutations affect vascular development. Because NEK promoters were active in xylem, phloem, and cambial regions, we investigated cotyledon vasculature, which provides a simple system for detecting defects in vein formation (Fig. 6). The *nek6-1* and *nek7-2* single mutants showed discontinuities in lateral veins, resulting in open vasculatures in the distal and middle regions of cotyledons (first and second loops, Fig. 6a and b). The phenotype was stronger in *nek6-1* than in *nek7-2*, and the *nek6-1 nek7-2* double mutant was almost identical to that of *nek6-1*, suggesting a major role of NEK6 in vascular development. These results indicate that *NEK* genes contribute to proper vascular pattern formation, consistent with their preferential expression in vascular tissues.

### Defects of flowering and inflorescence development of *nek* mutants

In addition to vascular tissues, several NEK genes showed promoter activity in the shoot apex and floral primordia, raising the possibility that NEKs participate in reproductive development. Therefore, we next examined flowering and inflorescence phenotypes of *nek* mutants. We found that the *nek123* triple mutant showed early flowering compared to the WT under long-day conditions (16-h light and 8-h dark cycles) (Fig. 7a). On the other hand, the *nek23456* quintuple mutant showed delayed flowering under the same long-day condition (Fig. 7b). In addition, the quintuple mutant exhibited aberrant inflorescence development (Fig. 7b). These results suggest that NEK1, NEK2, and NEK3 suppress floral transition, whereas NEK4, NEK5, and NEK6 promote flowering and proper inflorescence development. To examine whether NEKs act in the FLOWERING LOCUS T (FT)-dependent flowering pathway (Kardailsky et al. 1999, Kobayashi et al. 1999, Notaguchi et al. 2008), we analyzed the effect of *nek2-1, nek3-1*, and *nek4-1* mutations on the early flowering phenotype of *FD:FT* in the *ft-1* mutant. The *ft-1* mutant exhibits a severe late-flowering phenotype, which is masked by the ectopic expression of FT in the shoot apical meristem under the control of the *FD* promoter (Abe et al. 2005). The *nek2-1* and *nek3-1* mutations, individually or combinedly, promoted early flowering in the genetic background of *FD:FT* in *ft-1* (Fig. 7c). The *nek2-1 nek3-1* double mutant showed an early-flowering phenotype both with and without the *FD:FT* transgene in *ft-1* (Fig. 7c). The double-mutant phenotype seemed to be clear in the background of *FD:FT* in *ft-1*. The *nek4-1* mutation did not affect flowering with or without the *FD:FT* transgene in *ft-1*. These results suggest that NEK2 and NEK3 negatively regulate flowering, possibly through modulation of FT’s function.

### Interactions between NEKs and FT

Because the flowering phenotypes suggested that NEKs act in the FT-dependent flowering pathway, we examined the interaction between NEK proteins and FT. Because a tomato NEK homolog, SPAK, was previously reported to interact with FT and FT-related proteins such as SP (tomato CEN/TFL1 ortholog), we tested whether *Arabidopsis* NEKs interact with FT and TFL1 using yeast two-hybrid assays. TFL1 is a paralogous protein of FT, which suppresses floral transition antagonistically with FT (Kardailsky et al. 1999, Kobayashi et al. 1999). NEK2, 3, and 4 interacted with FT and TFL1 in yeast (Fig. 8a). We examined *in vivo* interaction of NEK4 with FT using bimolecular fluorescent complementation (BiFC). NEK4 fused with the N-terminal fragment of Yellow Fluorescent Protein (^N^YFP) and FT fused with the C-terminal fragment of YFP (^C^YFP) were coexpressed under the control of the cauliflower mosaic virus 35S RNA promoter in leaves of *Nicotiana benthamiana* by agroinfiltration. YFP fluorescence was recovered in the cytoplasm and nucleus, indicating interaction *in vivo* (Fig. 8b). We next tested whether NEKs phosphorylate FT and/or TFL1. We incubated recombinant maltose-binding protein (MBP)-tagged FT or TFL1 proteins with GST-NEK3 in the presence of [γ-^32^P]-ATP. FT was strongly phosphorylated by GST-NEK3, whereas phosphorylation of TFL1 was weak (Fig. 8c). Because FT interacts with the bZIP transcription factor FD to induce flowering (Abe et al. 2005), we examined whether NEK3 affects the FT–FD interaction using BiFC analysis. Coexpression of NEK3 reduced the BiFC signal between FT and FD in the nucleus (Fig. 8d and Supplementary Fig. S7), suggesting that NEK3 inhibits FT–FD complex formation, possibly through phosphorylation of FT.

**Figure 8:**
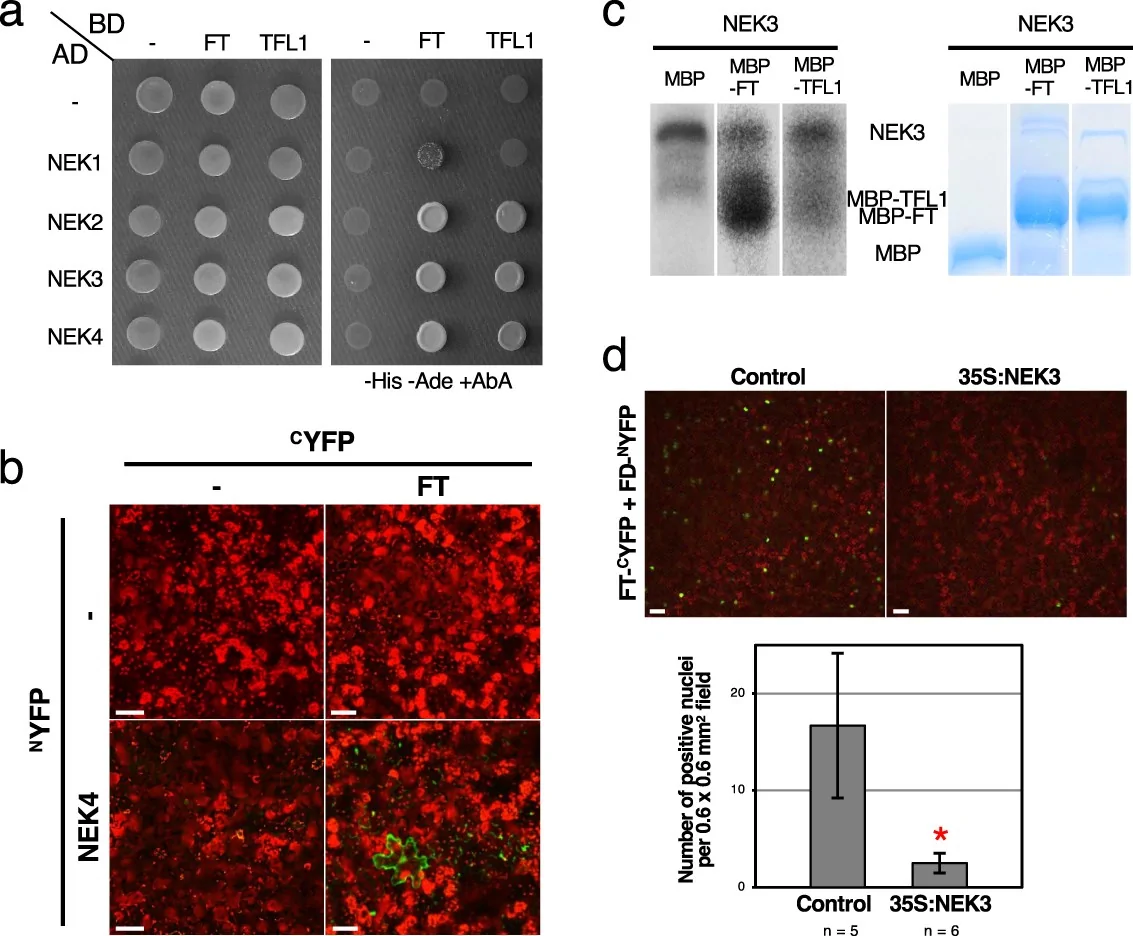
Interaction of NEKs with FT. (a) Interaction of NEKs with FT and TFL1 in the yeast two-hybrid assay. Yeast cells were cotransformed with the bait vector (BD-FT/TFL1) and prey vectors (C-terminal region of NEKs fused with GAL4-AD), plated on SD/–Leu/–Trp (left) or SD–His/–Ade/–Leu/–Trp plus Aureobasidin A (right, –His –Ade +AbA), and incubated for 3 days. (b) Interaction of NEK4 with FT in BiFC analysis. The ^C^YFP-tagged FT and ^N^YFP-tagged NEK4 were transiently expressed in leaves of *N. benthamiana* and leaf samples were observed under a confocal microscope. BiFC signals (green); chlorophyll autofluorescence (red). Bars = 50 *μ*m. (c) NEK3 preferentially phosphorylates FT *in vitro*. Recombinant GST-NEK3 was incubated with MBP and recombinant MBP-FT or MBP-TFL1 in the presence of [γ-^32^P]-ATP. Protein samples were separated by SDS-PAGE and subjected to autoradiography (left). Right-hand panel: Coomassie Brilliant Blue (CBB) staining. (d) NEK3 suppresses interaction of FT and FD in BiFC analysis. The ^C^YFP-tagged FT and ^N^YFP-tagged FD were transiently expressed with or without 35Spro:NEK3 in leaves of *N. benthamiana* and leaf samples were observed under a confocal microscope. BiFC signals (green); chlorophyll autofluorescence (red). The lower panel shows the quantification of nuclei with BiFC signals in five and six independent samples (*n*). Values are mean ± SE. An asterisk shows significant difference from the value of the control (Student's *t-*test, *P* < 0.02). Bars = 50 *μ*m.

### NEK6 and NEK3 phosphorylate β- and γ-tubulins

In addition to their role in flowering, NEKs have been implicated in microtubule-dependent growth control. Because NEK6 was previously shown to phosphorylate β-tubulin (Motose et al. 2011, Takatani et al. 2017), we next examined whether other NEK members share this biochemical activity. We analyzed the phosphorylation of tubulin proteins by NEK6 and other NEK members. We incubated recombinant His-tagged α-tubulin 4 (TUA4), β-tubulin 4 (TUB4), or γ-tubulin 2 (TUBG2) with GST-NEK6 in the presence of [γ-^32^P]-ATP (^32^P-labelled adenosine triphosphate). Both TUB4 and TUBG2 were strongly phosphorylated by GST-NEK6 (Fig. 9a). We also successfully purified recombinant active GST-NEK3 protein, which was subjected to the phosphorylation assay. We incubated recombinant His-tagged α-tubulin (TUA4, 6), β-tubulin (TUB4, 6), or γ-tubulin (TUBG2) with GST-NEK3 in the presence of [γ-^32^P]-ATP. GST-NEK3 preferentially phosphorylated β-tubulin and γ-tubulin (Fig. 9b). GST-NEK7 recombinant protein did not show any kinase activity using the above tubulin proteins and myelin basic protein as the substrates. These results indicate that NEK6 and NEK3 preferentially phosphorylate β- and γ-tubulin *in vitro*. These results suggest that NEK family members share kinase activity toward tubulin proteins, consistent with the defects in directional growth observed in *nek* mutants.

**Figure 9:**
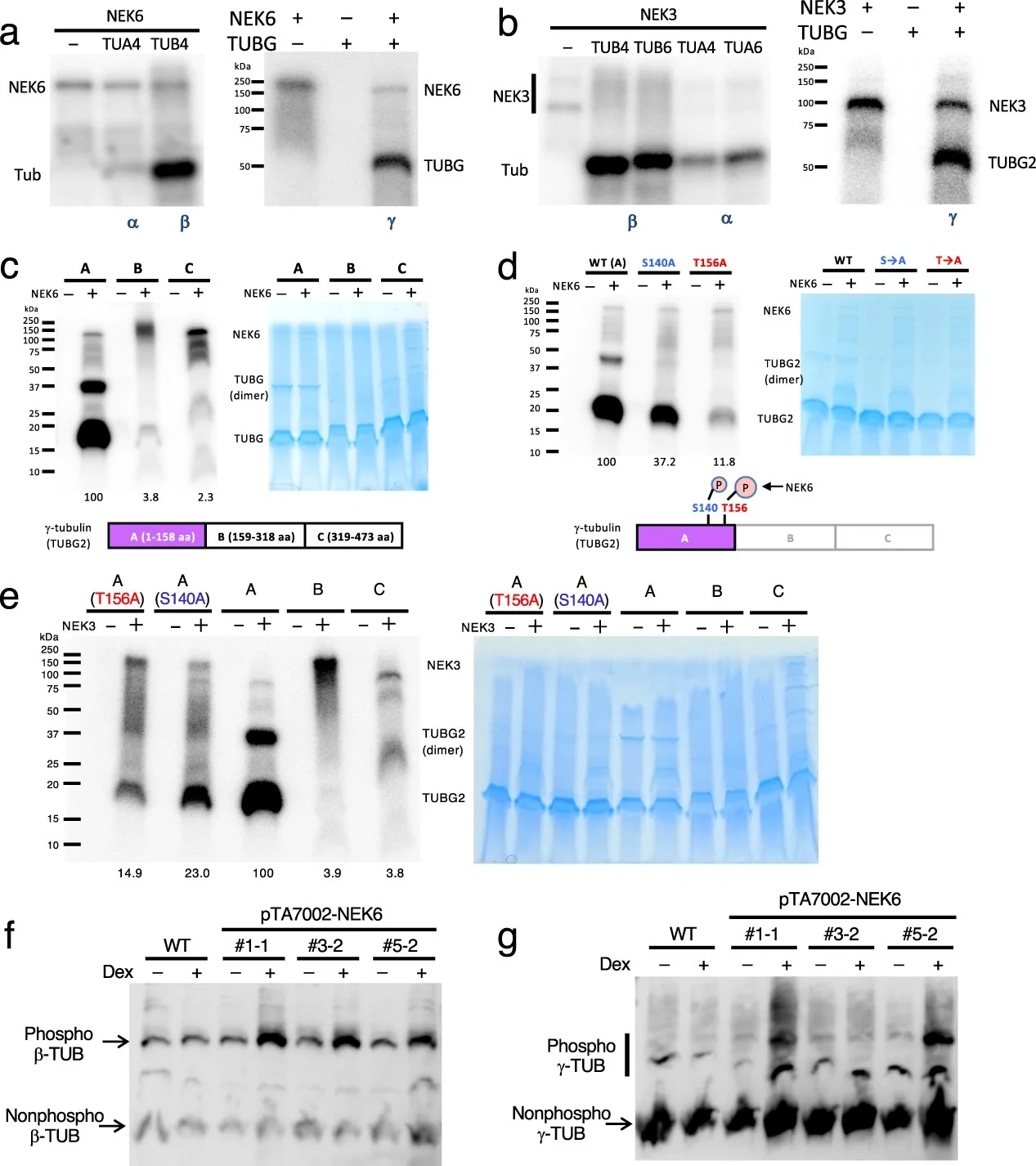
NEK3 and NEK6 phosphorylate beta- and gamma-tubulin. (a and b) Phosphorylation of tubulin by NEK6 and NEK3. Recombinant GST-NEK6 or GST-NEK3 was incubated with or without recombinant *Arabidopsis* β-tubulin (TUB4/6) or α-tubulin (TUA4/6) in the presence of [γ-^32^P]-ATP. Protein samples were separated by SDS-PAGE and subjected to autoradiography. (c–e) NEK6 and NEK3 preferentially phosphorylate T156 of TUBG2 (γ-tubulin). Recombinant TUBG2 fragments were incubated with or without recombinant GST-NEK6 or GST-NEK3 in the presence of [γ-^32^P]-ATP. Protein samples were separated by SDS-PAGE and subjected to autoradiography (left). Right-hand panels: CBB staining. (f and g) NEK6 phosphorylates β-tubulin and γ-tubulin *in vivo*. The WT and transgenic plants harboring the Dex-inducible NEK6 construct (pTA7002-NEK6) were treated with 30 *μ*M Dex for 24 h and protein samples were subjected to Phos-tag SDS-PAGE followed by immunoblotting with anti-β-tubulin or γ-tubulin antibodies.

### Identification of γ-tubulin phosphorylation sites

Having established that NEK3 and NEK6 phosphorylate γ-tubulin, we next sought to identify the phosphorylation sites (Fig. 9c–e). Three truncated TUBG2 constructs (His-tagged fragments A, B, and C) were incubated with GST-NEK6 or GST-NEK3 in the presence of [γ-^32^P]-ATP. Only the N-terminal fragment A was phosphorylated by NEK6 and NEK3 (Fig. 9c and e). We found two putative phosphorylation sites, S140 and T156, in fragment A, based on the consensus NEK phosphorylation motif (LxxS/T). These sites were substituted with nonphosphorylatable alanine residues and subjected to the phosphorylation assay. T156A substitution strongly reduced phosphorylation by NEK6 (~88% reduction) and NEK3 (~85% reduction), whereas S140A substitution resulted in ~63% and ~77% reduction of phosphorylation by NEK6 and NEK3, respectively (Fig. 9d and e). Thus, NEK6 and NEK3 phosphorylate both S140A and T156 of TUBG2 *in vitro*, with a preference for T156.

### *In vivo* phosphorylation of tubulin proteins by NEK6

We generated transgenic plants in which expression of *NEK6* was induced by the addition of dexamethasone (Dex). After the Dex treatment, total proteins were extracted and subjected to Phos-tag SDS-PAGE followed by immunoblotting with anti-tubulin antibodies. Phos-tag interacts with phosphorylated proteins and reduces their mobility during electrophoresis, which results in a band shift towards higher molecular weights on a gel. Dex-mediated induction of NEK6 increased the phosphorylated forms of both β- and γ-tubulin (Fig. 9f and g), indicating that NEK6 phosphorylates β- and γ-tubulin *in vivo*.

Taken together, our results demonstrate that plant NEKs have overlapping but distinct expression patterns, show partially redundant developmental functions, and share phosphorylation activity toward tubulin and FT proteins, suggesting that NEKs regulate organ growth and flowering through phosphorylation-dependent mechanisms.

## Discussion

In this study, we characterized the expression patterns of seven *NEK* genes in *A. thaliana*. Their distinct yet partially overlapping expression patterns suggest both functional diversity and redundancy among *NEK* family members. Notably, similarities and differences in their expression could not be predicted from the phylogenetic relationships of the *NEK* genes.

In the root apical meristem, promoter activity was detected for *NEK1, NEK2, NEK3, NEK4*, and *NEK6* genes, among which *NEK2, NEK3*, and *NEK6* showed particularly strong activity. These three genes were also active in the shoot apex, suggesting that they are likely involved in meristematic function. In the inflorescence, *NEK2, NEK3*, and *NEK6* were also expressed, with *NEK2* and *NEK3* showing higher promoter activity, consistent with their function in flowering described below.

In vascular tissues, *NEK1* and *NEK5* promoters drove expression in the xylem, while *NEK4, NEK6*, and *NEK7* promoters drove expression in the phloem. *NEK5, NEK6*, and *NEK7* also showed potent promoter activity in guard cells and their precursors. These results suggest that NEK genes are involved in the development and function of these specialized cell types.

To investigate the functions of the *Arabidopsis NEK* genes, we analyzed T-DNA insertion mutants. Single mutants showed no obvious phenotype except for *nek6* mutants, whereas multiple mutants exhibited clear defects in root growth direction, leaf elongation, and flowering. In multiple mutants, roots consistently grew toward the right, suggesting that *NEK* genes redundantly regulate the direction of root elongation. Mutants affecting microtubule function typically exhibit twisting of cell files (Furutani et al. 2000, Thitamadee et al. 2002, Yuen et al. 2003, Buschmann et al. 2004, Sedbrook et al. 2004, Ishida et al. 2007, Nakamura and Hashimoto 2009), whereas *nek* multiple mutants showed different defects, including disorganized cell-file alignment and reduced cell elongation. This suggests that the *NEK* genes function differently from other microtubule-associated proteins, such as SPIRAL1 and SPIRAL2.

During leaf morphogenesis, the *nek123* mutant displayed enhanced elongation of both petioles and leaf blades, whereas the *nek6* mutant showed reduced petiole elongation. Because the *nek123* phenotype is particularly pronounced in the petiole, *NEK1, NEK2*, and *NEK3* likely suppress petiole elongation, whereas *NEK6* promotes it. These antagonistic effects may regulate leaf elongation.

Previous studies have shown that leaf length and width are regulated independently through the actions of ROTUNDIFOLIA3 (*ROT3*) and ANGUSTIFOLIA genes, respectively (Tsuge et al. 1996). *ROT3* encodes a cytochrome P450 enzyme, CYP90C1, involved in brassinosteroid biosynthesis (Kim et al. 1998, 2005), indicating that brassinosteroids promote leaf elongation. Petiole elongation is also regulated by multiple factors, including auxin, brassinosteroids, cell wall properties, microtubules, light, and temperature (e.g. Kozuka et al. 2010, Sasidharan et al. 2014). Although the factors involved in NEK-mediated leaf elongation remain unclear, NEKs may regulate microtubule organization during leaf development, as shown for NEK6 in hypocotyl growth (Takatani et al. 2017, 2020). Because microtubule-mediated mechanical feedback is required for leaf morphogenesis (Zhao et al. 2020), altered responses of cortical microtubules to tension in *nek* mutants may underlie leaf growth phenotypes.

Furthermore, our results also suggest that NEK6 and NEK7 are required for continuous formation of leaf vasculature. Although the relationship between NEK6-mediated vein patterning and leaf elongation remains unclear, NEK6 may regulate both processes through microtubule-dependent control of cell elongation. While microtubules are known to regulate secondary cell wall formation in xylem vessels (Oda 2018), their role in leaf vascular patterning remains elusive. A microtubule-associated protein, CLASP (Cytoplasmic Linker protein-ASsociating Protein), regulates auxin transport by promoting recycling of PIN2 (Ambrose et al. 2013), but this mechanism may not apply to NEK6 because *nek6* mutants do not show typical auxin-related defects such as gravitropism. It will be interesting to test whether NEK6-mediated suppression of mechanical feedback also contributes to vascular development.

We further found that NEK3 and NEK6 phosphorylate both β-tubulin and γ-tubulin, suggesting that this activity contributes to the regulation of root growth direction and leaf elongation. NEK6-dependent phosphorylation of β-tubulin promotes depolymerization of cortical microtubules and regulates cell elongation (Takatani et al. 2017). Our results suggest that other NEK proteins also function as tubulin kinases that control microtubule organization.

We identified two phosphorylation sites in γ-tubulin, T156 and S140, targeted by NEKs. Although their functional significance remains unclear, these phosphorylation sites may regulate the γ-tubulin ring complex (γTuRC), which serves as a microtubule template that nucleates nascent microtubules (Kollman et al. 2011). Most cytoplasmic γTuRCs are inactive until recruited to existing microtubules (Murata et al. 2005, Chan et al. 2009), suggesting that their activity is regulated by associated proteins and/or posttranslational modifications (Kollman et al. 2011, Hashimoto 2015, Yagi et al. 2024). While phosphorylation of α- and β-tubulin has been reported to depolymerize cortical microtubules (Ban et al. 2013, Fujita et al. 2013, Takatani et al. 2017), γ-tubulin phosphorylation has been less studied in plants. Phosphorylation of γ-tubulin regulates microtubule assembly and spindle formation in budding yeast (Vogel et al. 2001, Keck et al. 2011, Lin et al. 2011, Peng et al. 2015). In addition, Human Synapses of Amphids Defective B (SADB) Ser/Thr kinase phosphorylates S131 of γ-tubulin to promote centrosome duplication (Alvarado-Kristensson et al. 2009). A casein kinase, Hrr25, of budding yeast phosphorylates S140 *in vitro* (Peng et al. 2015) but its biological function remains unknown. Because S140 is required for GTP binding, which is essential for γ-tubulin-mediated microtubule nucleation (Aldaz et al. 2005, Gombos et al. 2013), its phosphorylation may alter the affinity of γ-tubulin to GTP.

Our study suggests that T156 is a major phosphorylation site targeted by plant NEKs, though T156 phosphorylation is not reported previously. Structural modeling indicates that T156 is a surface residue proximal to Y161, which forms a hydrogen bond with E115 on helix 3 that is involved in the interaction between γ-tubulin molecules (Supplementary Fig. S8). Phosphorylated T156 may form a hydrogen bond with Y161 to suppress the Y161–E115 interaction, resulting in the exposure of E115 to the interface between γ-tubulin molecules. Thus, phosphorylation of T156 is assumed to suppress γ-tubulin interaction and microtubule nucleation. In summary, γ-tubulin phosphorylation by NEKs may function as the off-switch of microtubule nucleation to regulate microtubule assembly and organization.

We also found that *NEK* genes are required for reproductive development, particularly for floral transition and inflorescence development. The *nek123* mutant exhibited an early-flowering phenotype, whereas the *nek*23456 mutant showed delayed flowering. The *nek2* and *nek3* mutations promoted flowering even with ectopic *FT* expression in the shoot apical meristem by the *FD* promoter, which itself cause early flowering, whereas the *nek4* mutation had little effect. These results suggest opposing functions among *NEKs*: *NEK2* and *NEK3* suppress floral transition, whereas *NEK5* and *NEK6* promote flowering and/or inflorescence growth. This antagonistic relationship resembles their opposite effects on leaf elongation.

We further found that NEK2, NEK3, and NEK4 interact with FT, and our results suggest that NEK3 phosphorylates FT to inhibit its interaction with FD, possibly by interfering with its binding to 14-3-3 proteins, which mediate the FT–FD interaction (Taoka et al. 2011). Based on promoter activity, NEK2 and NEK3 may regulate FT function in the shoot apical meristem.

Previous studies have shown that *Arabidopsis* Calcium Dependent Protein Kinases (CDPKs), CPK6 and CPK33, phosphorylate T282 of FD near the C-terminus before reproductive transition (Kawamoto et al. 2015a, 2015b). Phosphorylation of T282 promotes the interaction of FD with FT via 14-3-3 proteins. Our study implies that phosphorylation of FT may suppress its interaction with FD. These opposing phosphorylation events could fine-tune floral transition. However, it should be noted that FT phosphorylation was detected *in vitro* and the effect of NEK3 on the FT–FD interaction was assessed in a transient assay in tobacco leaves. More detailed biochemical and genetic analyses are required to define NEK’s function in flowering. Future work should aim to identify the *in vivo* function of NEKs in flowering transition and elucidate the functional significance of FT phosphorylation.

In conclusion, NEK genes appear to redundantly regulate organ elongation through tubulin phosphorylation and control floral transition through FT phosphorylation. This dual function may be conserved in land plants because a bryophyte MpNEK (*Marchantia polymorpha* NEK) regulates tip growth of rhizoids and promotes the growth of reproductive organs (Otani et al. 2018, Mase et al. 2025). These functions would be assigned to the specific members of evolutionarily diverged NEKs in angiosperms. Further studies of the plant NEK family will provide new insights into phosphorylation-dependent coordination of vegetative and reproductive development.

## Materials and Methods

### Plant material and growth conditions

The *A. thaliana* Columbia (Col-0) accession was used as the WT for most experiments, while the Landsberg erecta and Wassilewskija accessions were used as control lines in experiments with *nek4-2* and *nek6-2*, respectively. The *ft-1* mutant with *FD:FT* was described previously (Abe et al. 2005). The T-DNA mutants *nek6-1* (*ibo1-4*, SALK_152782) and *nek6-2* (FLAG_397A06) were described previously (Motose et al. 2008, Sakai et al. 2008). *nek6-3* (*ibo1-1*), *nek6-4* (*ibo1-2*), *nek6-5* (*ibo1-3*), and *nek6-6* (*ibo1-5*) were isolated in the forward genetic mutant screening of ethyl methanesulfonate-mutagenized populations of *Arabidopsis* (Motose et al. 2008, 2012) The T-DNA mutants *nek2-1* (SALK_012284), *nek3-1* (SALK_056986), *nek4-1* (SALK_002006), *nek5-1* (SALK_104825), and *nek7-1* (SALK_134382) have been described previously (Motose et al. 2011). The other T-DNA mutants *nek1-1* (SALK_065629), *nek1-2* (SAIL_381_A06), *nek1-3* (SAIL_1271_G05), *nek1-4* (GK773C06), *nek2-2* (SALK_093269), *nek2-3* (SALK_144780), *nek2-4* (SALK_062288), *nek3-2* (SALK_127955), *nek3-3* (SALK_123055), *nek4-2* (GT_3_105710), *nek4-3* (SALK_200792), *nek4-4* (SK24824), *nek5-2* (SALK_142623), *nek5-3* (SALK_054652), *nek5-4* (SALK_058525), *nek5-5* (WiscDsLox412H03), *nek7-2* (SALK_127965), and *nek7-3* (SALK_024046) were obtained from the Arabidopsis Biological Resource Center (ABRC, Ohio State University, Columbus, Ohio, www.arabidopsis.org). Multiple mutants were generated by crosses of *nek1-1, nek2-1, nek3-1, nek4-1, nek5-2, nek6-4* (*ibo1-2*), and *nek7-1*. We could not obtain multiple mutants using the T-DNA mutant *nek6-1* due to the lack of recombination within chromosome 3 between *NEK5* and *NEK6* alleles and between *NEK6* and *NEK4* alleles. On the other hand, we could obtain the double mutants *nek2-1 nek6-1 and nek6-1 nek7-2* via recombination within chromosome 3 by crosses. The genotypes of mutants were confirmed by PCR with gene-specific primer sets (Supplementary Table S1).

Plants were grown on vermiculite or 1/2 Murashige and Skoog (MS) agar medium in the growth chambers under 16-h light/8-h dark conditions at 22°C. Before germination on the agar medium, seeds were surface-sterilized with a 1/4 bleach solution containing 0.01% (w/v) Triton X-100 for 5 min and were rinsed three times in sterile water. For the induction of NEK6, 7-day-old seedlings were treated with 30 *μ*M Dex in the MS liquid medium for 24 h.

### Plasmid construction and transformation

To construct promoter–GUS/GFP fusions, a ~2-kb promoter region upstream of the start codon of each *NEK* gene was amplified by PCR with gene-specific primer sets (Supplementary Table S1), cloned in pENTR/D-TOPO (Thermo), and transferred by LR reaction into the gateway cassette upstream of GUS and GFP in pGWB3 and pGWB4 (Nakagawa et al. 2007a), respectively. To construct NEK–GFP fusions expressed under the control of their own promoters, each gene promoter (~2 kb) and genomic coding sequence (CDS) before the stop codon was amplified by PCR with gene-specific primer sets (Supplementary Table S1), cloned in pENTR/D-TOPO (Thermo), and transferred by LR reaction into the gateway cassette upstream of GFP in pGWB550 (Nakagawa et al. 2007b). To construct glucocorticoid receptor–mediated NEK6 induction, full-length NEK6 was amplified by PCR with gene-specific primer sets (Supplementary Table S1) and cloned in pTA7002 (Aoyama and Chua 1997). The resulting constructs were introduced into the WT plants by the floral dip method (Clough and Bent 1998).

### Microscopy

Histochemical GUS staining was conducted under standard conditions (Jefferson et al. 1987). Technovit sections were prepared with a semimotorized rotary microtome (RM2245, Leica Microsystems, Germany) equipped with the tungsten carbide disposable blade TC-65 (Leica) as described previously. At least three independent transgenic lines were observed for GUS staining patterns using a light microscope DM5000B (Leica) equipped with a CCD camera DFC500 (Leica) or a light microscope E600 (Nikon, Japan) equipped with a CCD camera DP74 (Evident, Japan). For the analysis of cotyledon vasculature, 7-day-old seedlings grown on the agar medium were cleared in the mixture of chloral hydrate, glycerol, and water solution (8 g, 1 mL: 2 mL) and observed under a light microscope, DM5000B, equipped with a DFC500. To analyze the GFP signal, 7-day- or 10-day-old seedlings were placed on a slide glass (Matsunami) with small aliquots of water, covered with a glass strip [22 mm × 40 mm (or 24 mm × 36 mm), Thickness No. 1, Matsunami], and observed under a FV1200 confocal laser-scanning microscope (Evident) equipped with high-sensitivity GaAsP detectors and silicone oil objective lenses (30× and 60×, Evident) or a FV3000 confocal laser-scanning microscope (Evident) equipped with Gallium Arsenide Phosphide (GaAsP) detectors and a water objective lens (60×, Evident). Silicone oil (SIL300CS, Evident) or deionized water was used as immersion media for the objective lenses. At least three independent promoter–GFP transgenic lines were analyzed. BiFC was conducted as previously described (Abe et al. 2005) using a FV1200 or FV3000 confocal microscope. For observation of root cell files, the cell wall was stained with 1 mg/mL propidium iodide (Sigma-Aldrich) and observed by a confocal laser-scanning microscope FV1200 under the same conditions to compare the WT and mutants. The images were analyzed using ImageJ (National Institutes of Health, USA) and LPixel ImageJ plugins (LPixel, Japan).

### Y2H assay

Yeast two hybrid (Y2H) was performed using the Matchmaker Gold Yeast Two-Hybrid System (Clontech, CA, USA). The cDNAs encoding the full-length FT and TFL1 were amplified by PCR with gene-specific primer sets (Supplementary Table S1) and cloned in the *Sma* I site of pGBKT7 vectors, in which each gene was fused with the GAL4 DNA-binding domain. The pENTR/D-TOPO with the C-terminal-tail-coding regions of NEKs were generated from pENTR/D-TOPO with full-length NEK cDNAs (Motose et al. 2011) by removing the kinase-domain-coding regions with inverse PCR using a KOD-Plus Mutagenesis Kit (Toyobo) and primer sets (Supplementary Table S1). The C-terminal-tail sequences were transferred by LR reaction into a pGADT7-gateway vector (Sakai et al. 2008), in which each NEK C-terminal tail was fused with the GAL4 activation domain at the N-terminus. These vectors were introduced into the yeast strain Y2H Gold and analyzed on the basis of the activation of four marker genes, *HIS3, ADE2, MEL1*, and *AUR1-C*.

### RT-PCR

Total RNA was extracted with the NucleoSpin RNA Plant RNA isolation kit (Takara Bio, Kusatsu, Japan) according to the manufacturer’s instructions. The contaminated genomic DNA was digested with RNase-free DNase attached in the RNA isolation kit. RNA was quantified with NanoDrop (Beckman Coulter, CA, USA) and stored at −70°C. Reverse transcription was performed with 0.5 *μ*g of the total RNA of each sample by ReverTra Ace reverse transcriptase (Toyobo, Osaka, Japan) according to the manufacturer’s protocol. The synthesized first-strand cDNA was used as a template for PCR with KOD FX DNA polymerase (Toyobo) and gene-specific primer sets (Supplementary Table S1) for 28–30 cycles (NEKs) and 23 cycles (EF1a) according to the manufacturer’s protocol.

### *In vitro* Kinase assay

The cDNA encoding the full-length NEK3 was amplified by PCR with gene-specific primer sets (Supplementary Table S1), cloned in pENTR/D-TOPO entry vector, and transferred by LR reaction into the gateway cassette downstream of the GST-coding region in pDEST15 (Thermo). NEK6 was previously cloned in the GST fusion vector pGEX4T-2 (GE Healthcare) (Motose et al. 2008). The 6xHis-tagged TUB4/6 and TUA4/6 constructs were previously described (Motose et al. 2011, Takatani et al. 2017). The cDNA encoding the full-length TUBG2 was amplified by PCR with gene-specific primers (Supplementary Table S1), cloned into pENTR/D-TOPO, and transferred by LR reaction to the gateway cassette downstream of the 6×His tag coding region in pDEST17 (Thermo). The fragments and mutant versions of TUBG2 were generated from pDEST17-TUBG2 by inverse PCR using the KOD-Plus Mutagenesis Kit (Toyobo) and primer sets (Supplementary Table S1). The cDNAs encoding the full-length FT and TFL1 were amplified by PCR with gene-specific primer sets (Supplementary Table S1) and cloned between the *Xba* I and *Sal* I sites in the pMalc2 vector (New England Biolabs, NEB, MA, USA). Each construct was transformed into the *Escherichia coli* strain KRX (Promega). Recombinant GST-NEK proteins were expressed in the presence of 1 mM isopropyl 1-thio-beta-galactopyranoside at 16°C for 12 h and purified with glutathione–sepharose 4B (GE Healthcare) according to the manufacturers’ instructions. Recombinant 6×His-tagged tubulins and MBP-tagged proteins were induced by the addition of 0.2 mM isopropyl 1-thio-beta-galactopyranoside and 0.1% rhamnose and further incubated at 20°C for 16 h. The His-tagged tubulins were purified with the TALON metal affinity resin (Clontech) according to the manufacturer’s protocol. MBP-tagged FT/TFL1 were purified with maltose resin (NEB) according to the manufacturer’s method. One microgram of the GST-tagged recombinant proteins was incubated with or without 1 *μ*g His-tagged tubulin proteins or MBP-tagged proteins in 20 *μ*L of kinase buffer [50 mM Tris–HCl buffer (pH 7.4), 10 mM MnCl_2_, 1 mM dithiothreitol (DTT)] containing 10 *μ*Ci [γ-^32^P]-ATP at 24°C for 30 min. The reaction products were separated by sodium dodecyl sulfate-polyacrylamide gel electrophoresis (SDS-PAGE) and detected by autoradiography.

### Phos-tag SDS-PAGE and immunoblotting

Phosphorylated and nonphosphorylated proteins were separated by Phos-tag SDS-PAGE according to a standard method (Kinoshita and Kinoshita-Kikuta 2011). Seven-day-old seedlings treated with or without 30 *μ*M Dex were ground in liquid nitrogen and proteins were extracted in 50 mM Tris–HCl buffer (pH 7.4) containing 0.1% Triton X-100, Phosphatase Inhibitor Cocktail (Roche), and Complete Protease Inhibitor Cocktail (Roche). After centrifugation at 13 000 rpm for 10 min, the supernatants were used as crude protein extracts. Protein samples were precipitated with trichloroacetic acid and dissolved in SDS-PAGE sample buffer (1 mg/mL proteins) and then a 20 *μ*L aliquot of each sample was subjected to SDS-PAGE, in which 12.5 mM Phos-tag (Wako Pure Chemical) was added in the resolving polyacrylamide gel. After electrophoresis, gels were washed with Towbin buffer and proteins were transferred to polyvinylidene difluoride (PVDF) membranes (Bio-Rad) for immunoblotting with anti-beta-tubulin antibody (KMX-1, Millipore) or anti-gamma-tubulin rabbit polyclonal antibody generated against the purified 6xHis-tagged TUBG2.

### Phylogenetic analysis

For phylogenetic analysis, we used a conserved kinase domain. Sequence information was collected in Phytozome (http://phytozome.jgi.doe.gov/pz) and the 1KP database (https://db.cngb.org/onekp/). To generate an neighbor-Joining (NJ) bootstrap tree, the sequences were aligned by MUltiple Sequence Comparison by Log-Expectation (MUSCLE) and phylogenetic analyses were conducted in Molecular Evolutionary Genetics Analysis (MEGA7). The bootstrap consensus tree deduced from 1000 replicates was adopted to represent the evolutionary history of NEKs. The percentages of replicate trees in which the associated sequences clustered together are shown next to the branches. The branch lengths were measured in the number of substitutions per site.

### Statistical analyses

All the statistical analyses were conducted using R software (R 4.5.1, R Foundation for Statistical Computing, Vienna, Austria). The types of statistical tests, sample sizes, and significance levels (*P*-values) are indicated in the relevant figure legends (Figs. 4, 5, 7, 8, Supplementary Figs. S6, S7).

## Supplementary Material

Supplementary_materials_pcag056

## Data Availability

The data underlying this article will be shared on reasonable request to the corresponding author.
