# Supplementary material for: NIMA-related kinases redundantly regulate vegetative and reproductive development in Arabidopsis thaliana
Source: Plant Cell Physiol. 2026 Apr 28;67(10):1693–708. doi: 10.1093/pcp/pcag056 (PMC13714603; doi:10.1093/pcp/pcag056)
Supplement: Supplementary file 1 [file supplementary_materials_pcag056.zip › Table S1.pdf]

Table S1. Primers used in this study

Primer	Sequence	Purpose
NEK1 RT-PCR F	TGCCAAATATTCTGGTCCATTCCGA	RT-PCR
NEK1 RT-PCR R	TCAGAGGAGACCCACATGTGGTAT	RT-PCR
NEK2 RT-PCR F	ATGGAGAATTACGAGGTTCTTGAGCA	RT-PCR
NEK2 RT-PCR R	GTTGTGATGAAACAGGAAGTTGAC	RT-PCR
NEK3 RT-PCR F	GAAGGTCTGCTCATCAAGAGGGAAT	RT-PCR
NEK3 RT-PCR R	GATCCTCTGGACGATCCTTTC	RT-PCR
NEK4 RT-PCR F	GGCGAATCGTAGACGGGAAATTGT	RT-PCR
NEK4 RT-PCR R	GTTAAGCAGCTCGTTAGCGCTTG	RT-PCR
NEK5 RT-PCR F	AGTCCGAGAGACGAAAGTATGTGG	RT-PCR
NEK5 RT-PCR R	GGATGTCTTAGCAATTCTGCAGCAG	RT-PCR
NEK6 RT-PCR F	CCGGAGATCTGCTCATCAAGAGG	RT-PCR
NEK6 RT-PCR R	GGAGAAGAGTCAATCACTGGCATATGAT	RT-PCR
NEK7 RT-PCR F	ATGCAAATGGAGGCTAATGACTGTCA	RT-PCR
NEK7 RT-PCR R	GTTTCTTAACAATTCGCAGGCTGTTG	RT-PCR
NEK1-F(-2523)-cacc	caccAGCTCAGACCCTCCGGTGT	Cloning of the promoter & promoter-CDS in pENTR/D-TOPO
NEK1-R(-1)	TTCTAGCTAGCTTTGTTATTTTCTCACCTG	Cloning of the promoter in pENTR/D-TOPO
NEK1-F-cacc new	caccATGGAACAGTATGAGTTTCTTGAGCAAATTGG	Cloning of the CDS in pENTR/D-TOPO
NEK1-R-stop	TCAGAGGAGACCCACATGTGGTAT	Cloning of the CDS in pENTR/D-TOPO
NEK1-R-nonstop	GAGGAGACCCACATGTGGTATCTCT	Cloning of the promoter-CDS & CDS in pENTR/D-TOPO
NEK2-F(-2493)-cacc	caccCGACCACGGTGAAAAGACAAGACGA	Cloning of the promoter & promoter-CDS in pENTR/D-TOPO
NEK2-R(-1)	TGACAAACTCTGAAATTCACCTCCTTC	Cloning of the promoter in pENTR/D-TOPO
NEK2-F-cacc	caccATGGAGAATTACGAGGTTCTTGAGCA	Cloning of the CDS in pENTR/D-TOPO
NEK2-R-stop	TCAATCTCCCAGCTTAGTAGTAGTGG	Cloning of the CDS in pENTR/D-TOPO
NEK2-R-nonstop	ATCTCCCAGCTTAGTAGTAGTGGTTTC	Cloning of the promoter-CDS & CDS in pENTR/D-TOPO
NEK3-F(-2502)-cacc new	caccCGTACAAGGCATAATGTCGAGACATC	Cloning of the promoter & promoter-CDS in pENTR/D-TOPO
NEK3-R(-1)	CGAAAACAACCTCCTTAAAAATATTCTCTTCC	Cloning of the promoter in pENTR/D-TOPO
NEK3-F-cacc	caccATGGAGCATTACGAGGTTCTTGAGC	Cloning of the CDS in pENTR/D-TOPO
NEK3-R-stop	TCAATCTTCTTCCAAGTTACTAGGACTAC	Cloning of the CDS in pENTR/D-TOPO
NEK3-R-nonstop	ATCTTCTTCCAAGTTACTAGGACTACtttc	Cloning of the promoter-CDS & CDS in pENTR/D-TOPO
NEK4-F(-2070)-cacc	caccTCCTGGTCACACCAGTCATCTGA	Cloning of the promoter & promoter-CDS in pENTR/D-TOPO
NEK4-R(-1)	CTGACAAACACACTTGCTTGAAATTTC	Cloning of the promoter in pENTR/D-TOPO
NEK4-F-cacc	caccATGGAGCGGTATGAAGTCTTGGAG	Cloning of the CDS in pENTR/D-TOPO
NEK4-R-stop	CTACACGGAAGCTTCTTTGAAGCTC	Cloning of the CDS in pENTR/D-TOPO
NEK4-R-nonstop	CACGGAAGCTTCTTTGAAGCTCTTGGTC	Cloning of the promoter-CDS & CDS in pENTR/D-TOPO
NEK5-F(-3000)-cacc	caccACAATGGAGG CTCTAGTGGG AGCT	Cloning of the promoter & promoter-CDS in pENTR/D-TOPO
NEK5-R(-1)	CTGAGATCTTCCTCTGTCGGTTTAAAAG	Cloning of the promoter in pENTR/D-TOPO
NEK5-F-cacc new	caccATGGCAAACAAGATAAGTGAAACTGCTTCCA	Cloning of the CDS in pENTR/D-TOPO
NEK5-R-nonstop	AATATTGGTTTCATCATCGTTTTGCTTTCTCT	Cloning of the promoter-CDS & CDS in pENTR/D-TOPO
IBO1-F(-2104)-cacc	caccGCCGCAACGACTTACGTTTACTGC	Cloning of the promoter & promoter-CDS in pENTR/D-TOPO
IBO1-R(-1)	CGCTTTACCAAAAACCAACAACGAAACTTC	Cloning of the promoter in pENTR/D-TOPO
NEK7-F(-2501)-cacc new	caccCCGGTATTGTCATTCCGACCATCG	Cloning of the promoter & promoter-CDS in pENTR/D-TOPO
NEK7-R(-1)	GAAATCTTCAAAAAAGAAAAACCTTTCCCGG	Cloning of the promoter in pENTR/D-TOPO
NEK7-F-cacc	caccATGCAAATGGAGGCTAATGACTGTCA	Cloning of the CDS in pENTR/D-TOPO
NEK7-R-stop	TCAGGTTCCTTGGTTGAGTTTGTCAG	Cloning of the CDS in pENTR/D-TOPO
NEK7-R-nonstop	GGTTCCTTGGTTGAGTTTGTCAGac	Cloning of the promoter-CDS & CDS in pENTR/D-TOPO
NEK1-F-Ctail	CAGCCTTATGTTCTGGATGTCAAGC	pENTR-NEK C-tail (removal of kinase domain)
NEK2-F-Ctail	CAACCGTATATCCAGAAGATTCATTTG	pENTR-NEK C-tail (removal of kinase domain)
NEK3-F-Ctail	CAGCCCTACGTTCAAAAGGTTCTTC	pENTR-NEK C-tail (removal of kinase domain)
NEK4-F-Ctail	CAGCCTTACATCAGTATGGTTTATATG	pENTR-NEK C-tail (removal of kinase domain)
NEK5-F-Ctail	CAGCCATATCTTGCACAATGCCAAAATC	pENTR-NEK C-tail (removal of kinase domain)
NEK6-F-Ctail	CAGCCATATGTTGAGCAGTACCGTCCA	pENTR-NEK C-tail (removal of kinase domain)
NEK7-F-Ctail	CAACCATACCTTCTTCAATGCCAAAACC	pENTR-NEK C-tail (removal of kinase domain)
SALK_065629 LP	AATTGTCTTTGTCTCCCCACC	nek1-1 (SALK_065629) genotyping
SALK_065629 RP	ACACTAATGACCATGGTTGCC	
SAIL_381_A06 LP	ACGCTACGATCCAATTCACAC	nek1-2 (SAIL_381_A06) genotyping
SAIL_381_A06 RP	TATCTCGAAAATGCGTCATCC	
SAIL1271G05 LP	TTGGTTGCTTAAAATCCGATG	nek1-3 (SAIL_1271_G05) genotyping
SAIL1271G05 RP	TATGCCTTACCTTTTCCACCC	
GK773C06 LP	ACAGAGGATGTGAGATCGTCG	nek1-4 (GK773C06) genotyping
GK773C06 RP	TGGAAAAGGATCTTTTGGGTC	
SALK_012284 LP	TCATTCGTTTTCTTCCAATGC	nek2-1 (SALK_012284) genotyping
SALK_012284 RP	CCAAATTTGCAAATGAATTGG	
SALK_093269 LP	CTCCGGAAAAATCCAGAACTC	nek2-2 (SALK_093269) genotyping
SALK_093269 RP	CATACACCGCCTTCATAATCG	
SALK_144780 LP	GTCTCCTCCTTTGCAATAGCC	nek2-3 (SALK_144780) genotyping
SALK_144780 RP	TCTCCTAAATCTGCCGATTTG	
SALK_062288 LP	AAAGCAGGTTTCATAGCCGTC	nek2-4 (SALK_062288) genotyping
SALK_062288 RP	TCGTTAAGCCAATTCATTTGC	
SALK_056986 LP	GATCCTCTGGACGATCCTTTC	nek3-1 (SALK_056986) genotyping
SALK_056986 RP	CGCTGGGTAAGTTTCTCTTCC	
SALK_127955 LP	TTCCACAATAAATGGATTCCTTATC	nek3-2 (SALK_127955) genotyping
SALK_127955 RP	CTCCTCCCGAAAATGCTAAAC	
SALK_123055 LP	TGTCGCTCTGGTGTATGTTTG	nek3-3 (SALK_123055) genotyping
SALK_123055 RP	CCGGTTTATCCTGTTGATCAG	
SALK_002006 LP	TTATCAGTTTTGCCCAACTCG	nek4-1 (SALK_002006) genotyping
SALK_002006 RP	TGCGGATATGAATCCGATAAG	
GT_3_105710 LP	GGAATTGAGCAAGGAAAATCTC	nek4-2 (GT_3_105710) genotyping
GT_3_105710 RP	ACTCGACAACAAATGGATTGC	
SALK_200792C LP	GTGTCGGTACGATTGAAGCTC	nek4-3 (SALK_200792) genotyping
SALK_200792C RP	TGGGTGAGAGTGAGACAAACC	
SK24824 LP	TGGCTCTACGTTACTTAAGCGAG	nek4-4 (SK24824) genotyping
SK24824 RP	ACTCGACAACAAATGGATTGC	
SALK_104825 LP	CAAAGATTCCAATGCTTCTGC	nek5-1 (SALK_104825) genotyping
SALK_104825 RP	AGAGAACGAGGTTCGGTTAGG	
SALK_142623 LP	CAAAGATTCCAATGCTTCTGC	nek5-2 (SALK_142623) genotyping
SALK_142623 RP	AGAGAACGAGGTTCGGTTAGG	
SALK_054652 LP	CGGTACAGATTTTCTTTGGTTG	nek5-3 (SALK_054652) genotyping
SALK_054652 RP	GCCGCTAGATAACGAGCTTTG	
SALK_058525 LP	TCGAAAATGTTGTGGTGTTAGAG	nek5-4 (SALK_058525) genotyping
SALK_058525 RP	CTTTCGTCTCTCGGACTTGTG	
WiscDsLox412H03 LP	CGGTACAGATTTTCTTTGGTTG	nek5-5 (WiscDsLox412H03) genotyping
WiscDsLox412H03 RP	GCCGCTAGATAACGAGCTTTG	
nek6-1 LP	CTTTGAAAGCAGGTCGATACG	nek6-1 (ibo1-4, SALK_152782) genotyping
nek6-1 RP	TTTGGTTACAGGGCTGAGTTG	
FLAG_397A06 LP	CATAGCAACCCTGCATTTTTC	nek6-2 (FLAG_397A06) genotyping
FLAG_397A06 RP	TTGCTTACAACAGATTTGATTTG	
ibo1-dCAPS-F3	GGATTGCTCTCAAGCTTCTGAAC	nek6-3 (ibo1-1) genotyping (WT: *Sau* 3A I/Mbo I degradation)
ibo1-dCAPS-R	AACCATAAGGGATATCAGCAAGCAGaT	
ibo1-2 CAPS-F	CAGAGCCAAAGAGAACCTCTGTTGG	nek6-4 (ibo1-2) genotyping (WT: *Afa* I degradation)
ibo1-2 CAPS-R5	CTCAAGAGCTGAGTTCTGAGAC	
ibo1-3 dCAPS-F	AAGTCGTTCAGACAGAGAGCAGAAGC	nek6-5 (ibo1-3) genotyping (WT: *Pvu* II degradation)
ibo1-3 dCAPS-R	TTTCTCGAGGCGAAACTTTGTTCTTCCCAAACA	
SALK_024046 LP	CTATGAGGGAGGGAACATGTG	nek7-1 (SALK_024046) genotyping
SALK_024046 RP	TTGATGAGTCCAGCCATATCC	
SALK_127965 LP	GGTTGGCACAATTGTTACTGG	nek7-2 (SALK_127965) genotyping
SALK_127965 RP	TACTGTTGGCCGATATTCTGG	
SALK_134382 LP	GGTTGGCACAATTGTTACTGG	nek7-3 (SALK_134382) genotyping
SALK_134382 RP	TACTGTTGGCCGATATTCTGG	
FT-F-pGBKT7	ggaggccgaattcccgATGTCTATAAATATAAGAGACCCTC	InFusion cloning to S*ma* I site of pGBKT7 (Y2H)
FT-R-pGBKT7	AGGTCGACGGATcccCTAAAGTCTTCTTCCTCCGCAGCCA	InFusion cloning to S*ma* I site of pGBKT7 (Y2H)
TFL1-F-pGBKT7	ggaggccgaattcccgATGGAGAATATGGGAACTAGAGTGA	InFusion cloning to S*ma* I site of pGBKT7 (Y2H)
TFL1-R-pGBKT7	AGGTCGACGGATcccCTAGCGTTTGCGTGCAGCGGTTTCT	InFusion cloning to S*ma* I site of pGBKT7 (Y2H)
FT-F1-XbaI	AAATCTAGAATGTCTATAAATATAAGAGACCCTC	Cloning to pMalc2
FT-R-stop-SalI	AAAGTCGACCTAAAGTCTTCTTCCTCCGCAGCCA	Cloning to pMalc2
TFL1-F1-XbaI	AAATCTAGAATGGAGAATATGGGAACTAGAGTGA	Cloning to pMalc2
TFL1-R-stop-SalI	AAAGTCGACCTAGCGTTTGCGTGCAGCGGTTTCT	Cloning to pMalc2
NEK4-F-start-XhoI	aaactcgagATGGAGCGGTATGAAGTCTTG	Cloning to BiFC vector
NEK4-R-V555-KpnI	aaaggtaccCACGGAAGCTTCTTTGAAGC	Cloning to BiFC vector
TUBG2-F-start+cacc	caccATGCCGAGGGAGATAATTACGCT	Cloning in pENTR/D-TOPO
TUBG2-R-nonstop	GAACGCTAACTTAGGATCCGCAA	Cloning in pENTR/D-TOPO
TUBG2-R-474+stop	TCAATTCAAAGTCTCCAACAAATAAGA	Inverse PCR for fragment A from the full length
TUBG2-F-475	GATCGTTATAGCAAGAAGTTGGT	Inverse PCR for fragment B from fragment AB
TUBG2-R-954+stop	TCACTTTGCCTGACTAGCTTCTTTG	Inverse PCR for fragment AB from the full length
TUBG2-F-955	TACATATCAATATTGAATATAATTCA	Inverse PCR for fragment C from the full length
TUBG2-R-H139	GTGGCAAAGAACAAAACCCTC	Inverse PCR for the S140A substitution
TUBG2-F-S140A	gCTATTGCTGGAGGCACTGGC	Inverse PCR for the S140A substitution
TUBG2-R-E157	CTCCAACAAATAAGATCCCAT	Inverse PCR for the T158A substitution
TUBG2-F-T158A-DEST-A	gCTTTGAATTGAacccagct	Inverse PCR for the T158A substitution
TUBG2-F-T158A	gCTTTGAATGATCGTTATAGCAAG	Inverse PCR for the T158A substitution
pENTR-F1	AAGGGTGGGCGCGCCGACCCA	Innverse PCR
pENTR-R1	GGTGAAGGGGGCGGCCGCGGA	Innverse PCR
ENTR-D-F(+stop)	TGAAAGGGTGGGCGCGCCGAC	Innverse PCR
ENTR-D-R(+start)	CATGGTGAAGGGGGCGGCCG	Innverse PCR
