# Supplementary material for: NIMA-related kinases redundantly regulate vegetative and reproductive development in Arabidopsis thaliana
Source: Plant Cell Physiol. 2026 Apr 28;67(10):1693–708. doi: 10.1093/pcp/pcag056 (PMC13714603; doi:10.1093/pcp/pcag056)
Supplement: Supplementary file 1 [file supplementary_materials_pcag056.zip › Suppl Figs .pdf]

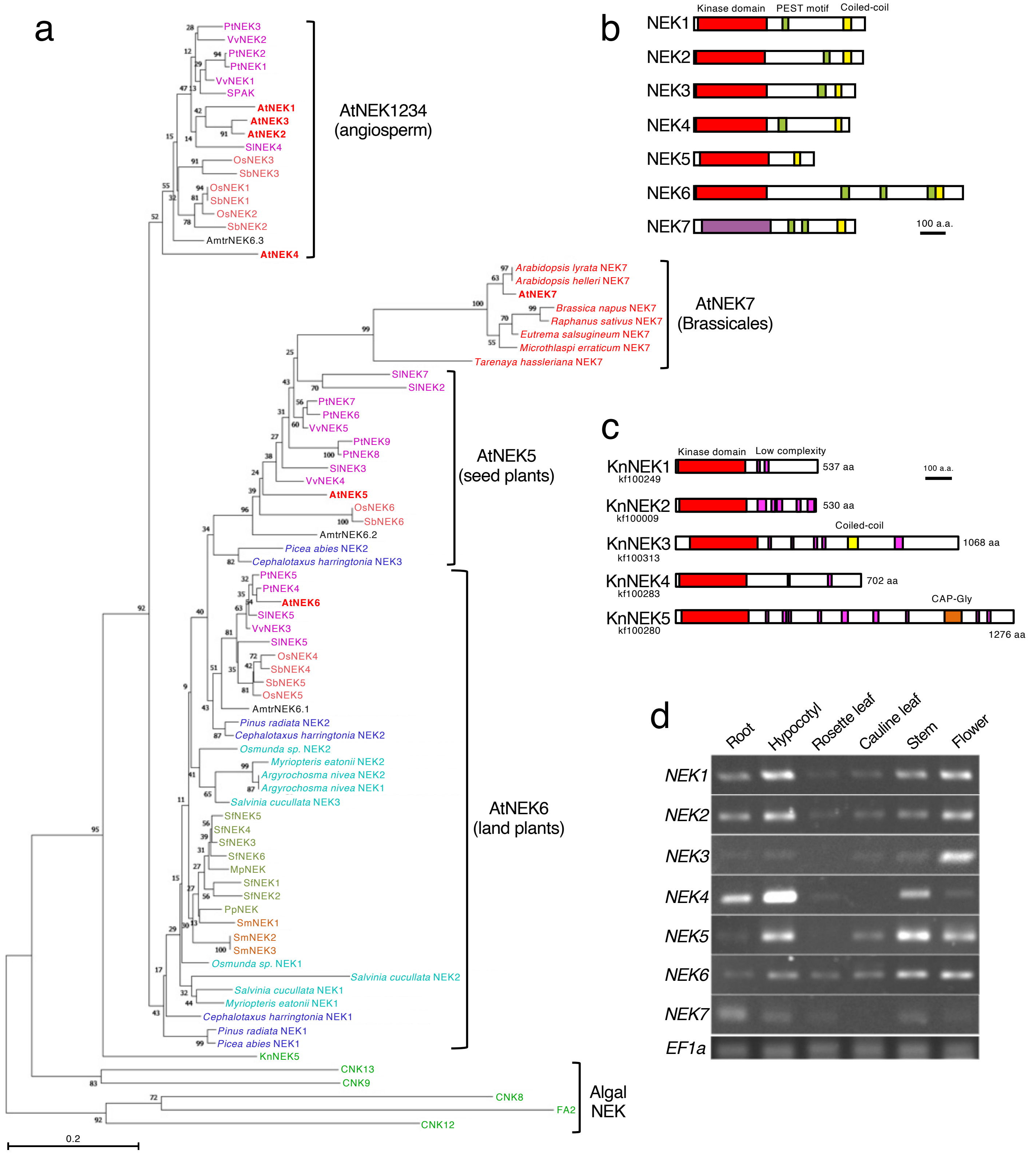


Supplementary Fig. S1. Structure and phylogenetic tree of NEKs.

(a) Phylogenetic tree of plant NEKs constructed by the neighbor joining method using the kinase domain (corresponds to the amino acid residues 8-262 of NEK6). Numbers on the branch nodes show bootstrap support values calculated from 1000 replicates. Amtr: *Amborella trichopoda*, At: *Arabidopsis thaliana*, Kn: *Klebsormidium nitens*, Mp: *Marchantia polymorpha*, Os: *Oryza sativa*, Pp: *Physcomitrium patens*, Pt: *Populus trichocarpa*, Sb: *Sorghum bicolor*, Sf: *Sphagnum fallax*, Sl: *Solanum lycopersicum*, Sm: *Selaginella moellendorffii*, Vv: *Vitis vinifera*. The names of species of ferns, gymnosperms, and Brassicales are written in the full words instead of the abbreviation. In Brassicales except *A. thaliana*, only NEK7 family is listed for the simplification in the limited space. For the same reason, algal NEKs are omitted except sister clade members (KnNEK5, CNK9/13) and typical representative NEKs (FA2, CNK8, CNK12). CNKs are *Chlamydomonas reinhardtii* NEKs. Green: alga, moss green: bryophytes, brown: lycophyte, light blue: monilophytes, blue: gymnosperms, black: basal angiosperm, orange: monocot, purple: dicot, red: Brassicales.

(b) Structure of NEKs in *A. thaliana*. Red: kinase domain, purple: dual kinase domain, green: PEST sequence, yellow: coiled-coil motif.

(c) Structure of NEKs in *K. nitens*. Red: kinase domain, pink: low complexity region, yellow: coiled-coil motif, brown: CAP-Gly domain.

(d) RT-PCR analysis of *NEK* genes. Total RNA was isolated in each organs shown above and subjected to reverse transcription followed by PCR using gene specific primers. *EF1a* is a loading control.

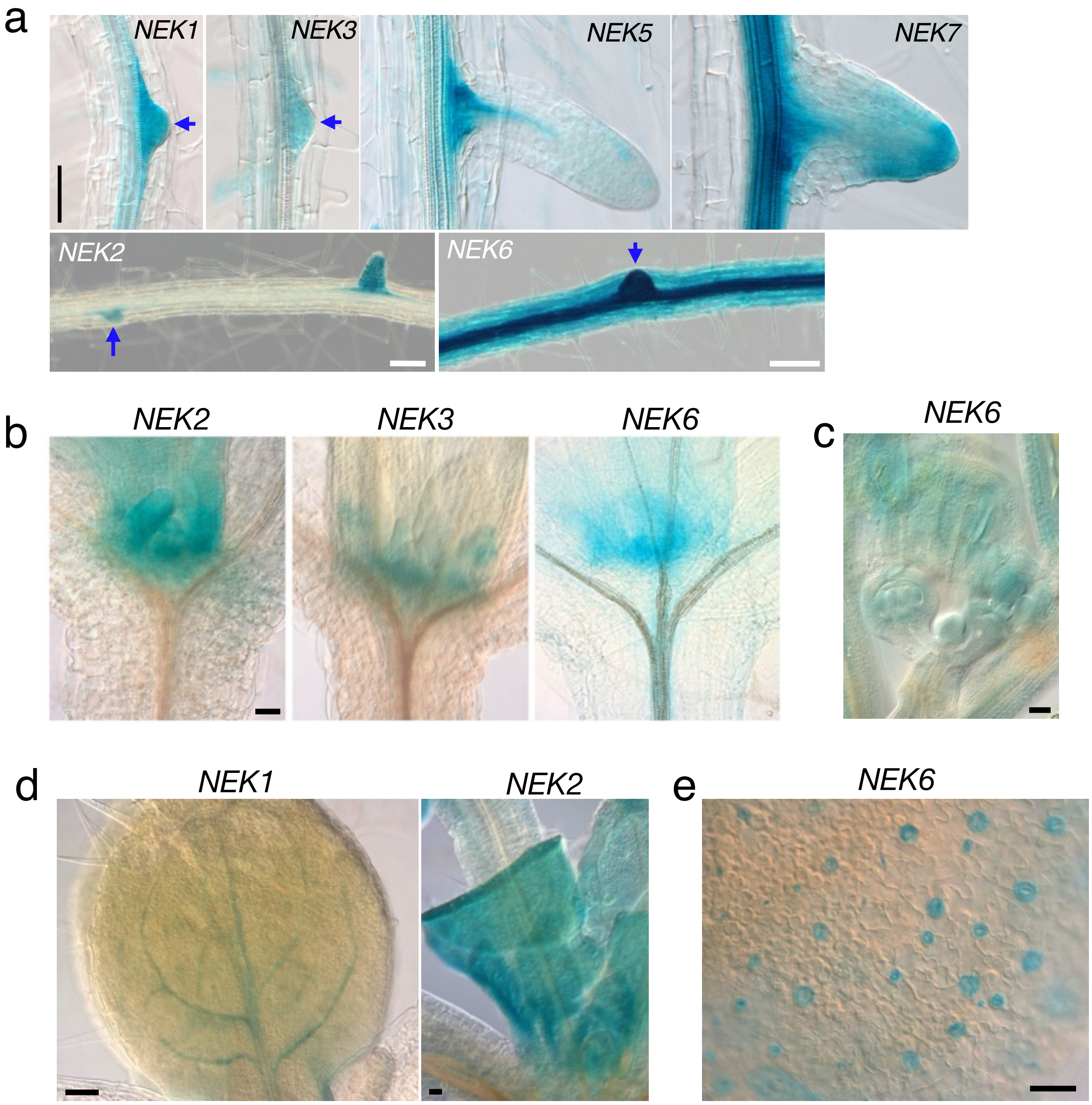


Supplementary Fig. S2. The promoter activity of *NEK* genes.
Histochemical GUS staining of *NEK* promoter-GUS fusions in lateral root primordia (a), shoot apex (b), inflorescences (c), leaves (d) and guard cells (e). Blue arrow in (a): lateral root primordium. Bars = 50 µm in (b, d, e) and 100 µm (a, c).

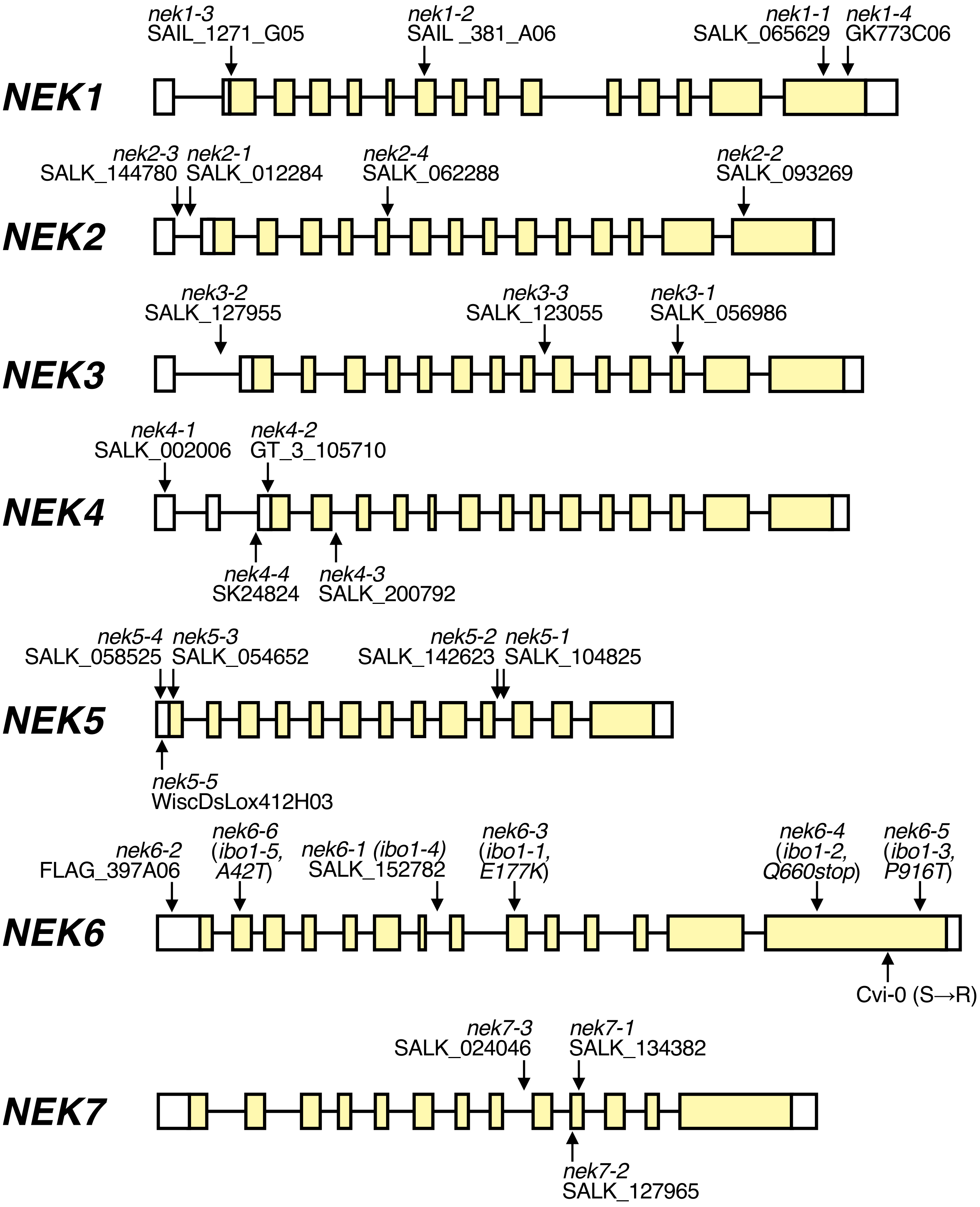


Supplementary Fig. S3. The mutants of *NEK* genes.
Arrows indicate the positions of T-DNA insertions or single base-substitution mutations. Boxes and lines show exons and introns, respectively. White box: 5'-UTR or 3'-UTR, yellow box: coding region.

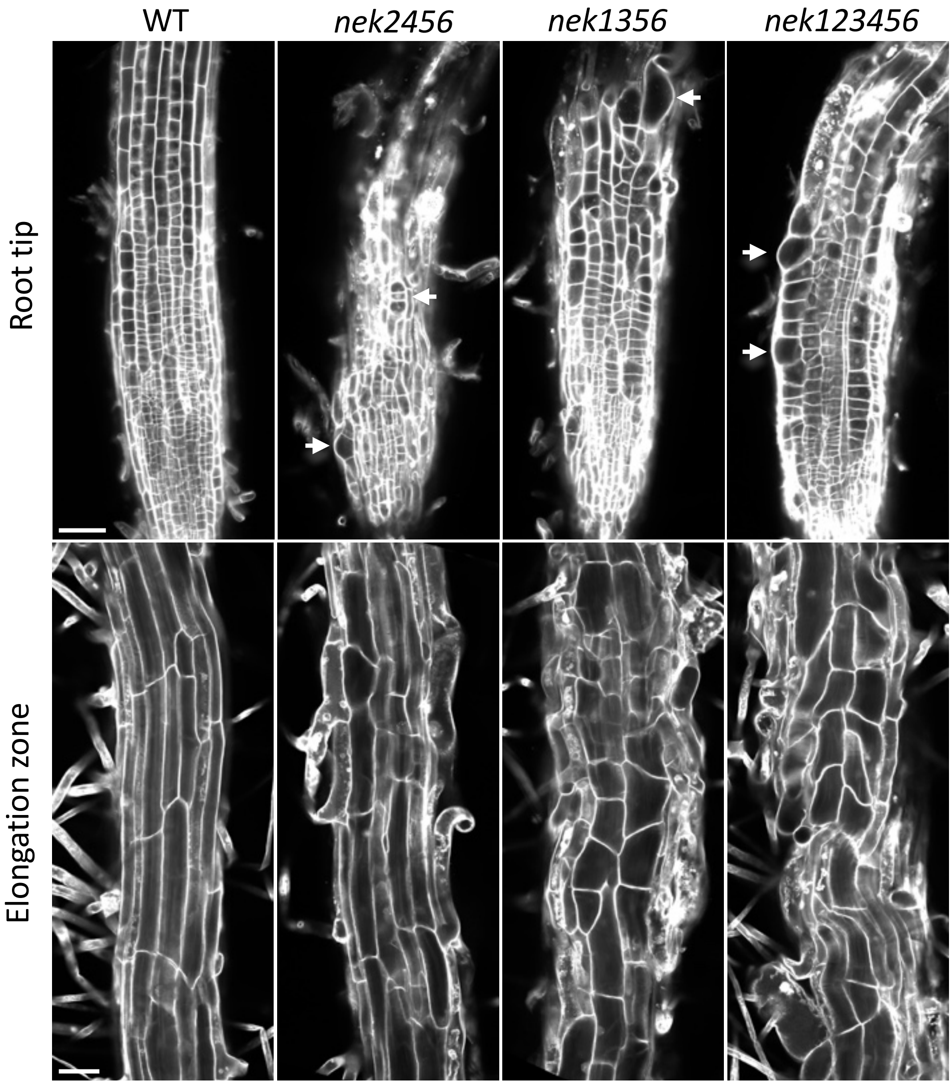


Supplementary Fig. S4. Aberrant cell growth and cell file formation in the *nek* multiple mutants. The wild type and the *nek* multiple mutants grown for 5 days on the agar medium were stained with PI and the root tip (a) and elongation zone (b) were observed under a confocal laser-scanning microscope. Arrows indicate aberrant cell swelling in root tips. Bars = 50 µm.

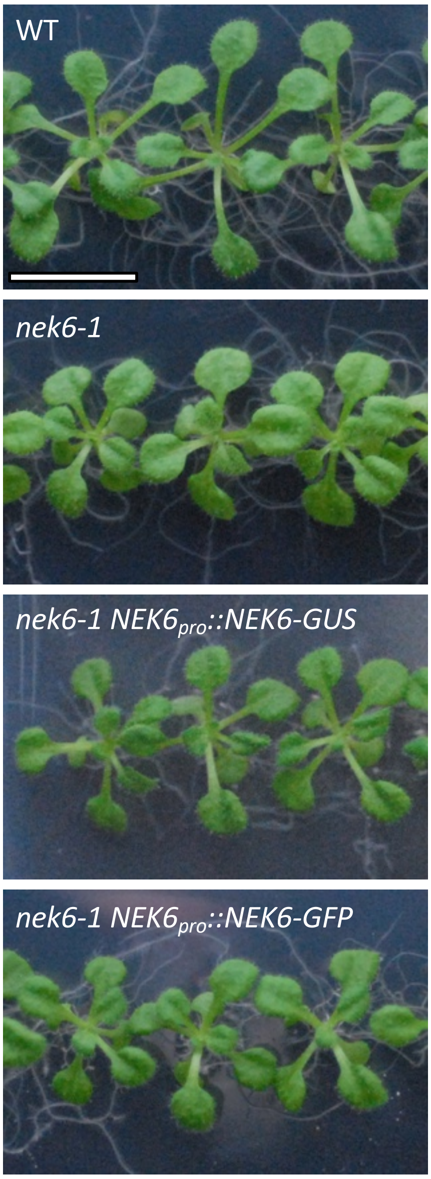


Supplementary Fig. S5. The morphology of the wild type, *nek6-1* mutant, and *nek6-1* mutant complimented with *NEK6pro:NEK6-GUS* or *NEK6pro:NEK6-GFP* grown on the ½ MS agar medium for two weeks.

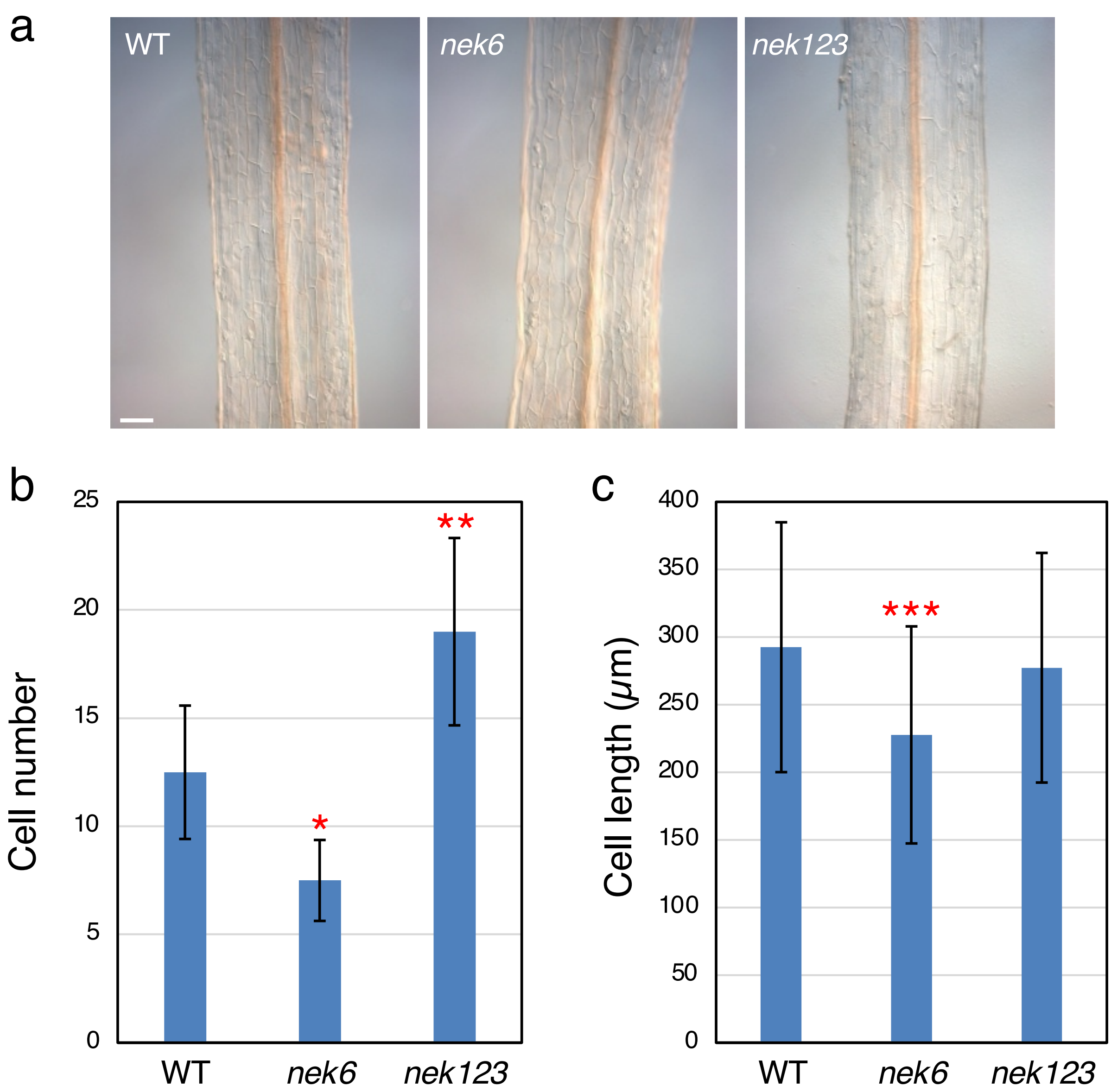


Supplementary Fig. S6. Number and length of epidermal cells in petioles.
(a) Petioles of first leaves in the wild type, *nek6* mutant, and *nek1 nek2 nek3* triple mutants grown on the ½ MS medium for one week. Bar = 100 µm.
(b) Number and (c) length of epidermal cells in the midribs of petioles of first leaves in the wild type, *nek6* mutant, and *nek1 nek2 nek3* triple mutants grown on the ½ MS medium for one week. The marked values show significant difference from the wild type values (Dunnett's test, *$P$ < 0.02, **$P$ < 0.005, ***$P$ < 0.0005, n = 8 plants in b, n = 48-60 cells in c).

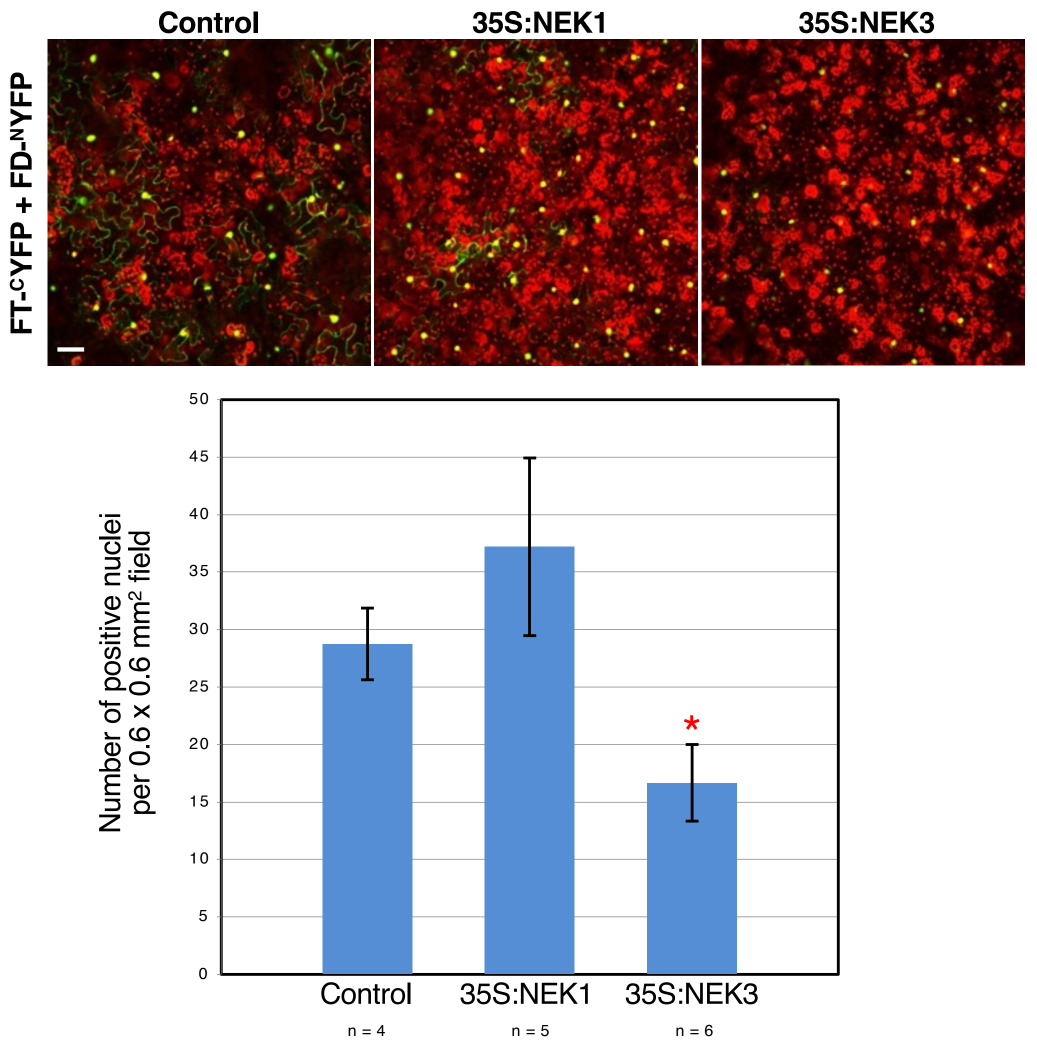


Supplementary Fig. S7. NEK3 suppresses interaction of FT and FD in BiFC analysis.
The $^{C}$YFP-tagged FT and $^{N}$YFP-tagged FD were transiently expressed with or without 35Spro:NEK1 or NEK3 in leaves of *N. benthamiana* and leaf samples were observed under a confocal microscope. BiFC signals (green), chlorophyll autofluorescence (red). Lower panel shows quantification of nuclei with BiFC signals in the independent samples (n = number of samples). Values are mean ± SE. An asterisk shows significant difference from the value of the control (Student's *t*-test, $P < 0.03$). Bars = 50 µm.

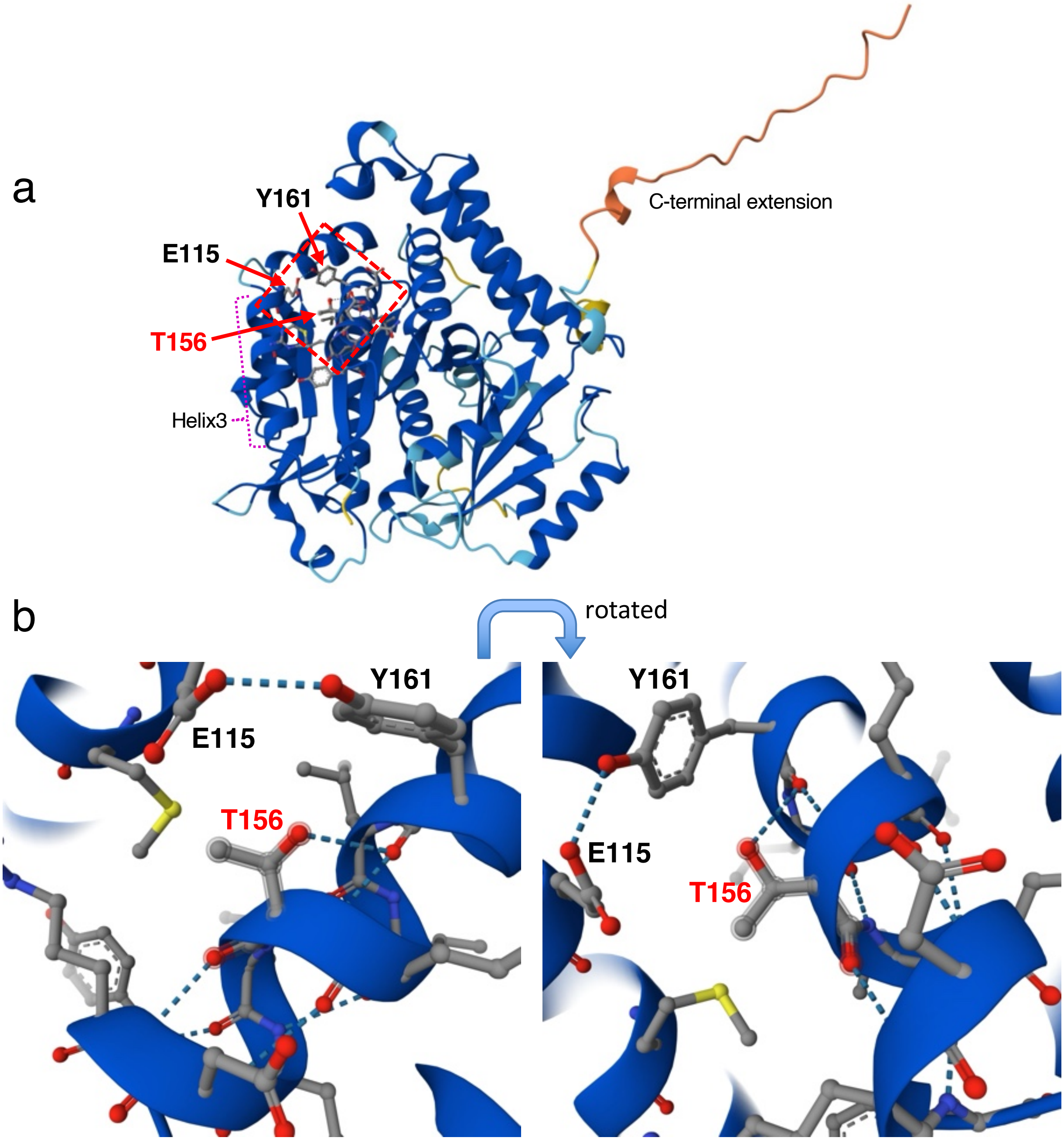


Supplementary Fig. S8. AlphaFold model of Arabidopsis TUBG2 ($\gamma$-tubulin).

(a) A whole 3D structure of TUBG2. Nearly minus-end view to show the spatial relationships of E115, T156, Y161. T156 is a phosphorylation target of NEKs.

(b) Enlarged view in the broken-lined square in (a). Red: oxygen, blue: nitrogen, yellow: sulfur, grey: carbon.
